# RecA Regulation by RecU and DprA During *Bacillus subtilis* Natural Plasmid Transformation

**DOI:** 10.3389/fmicb.2018.01514

**Published:** 2018-07-11

**Authors:** Ester Serrano, Begoña Carrasco, Jamie L. Gilmore, Kunio Takeyasu, Juan C. Alonso

**Affiliations:** ^1^Department of Microbial Biotechnology, Centro Nacional de Biotecnología – Consejo Superior de Investigaciones Científicas, Madrid, Spain; ^2^Graduate School of Biostudies, Kyoto University, Kyoto, Japan

**Keywords:** horizontal gene transfer, DNA strand exchange, plasmid transformation, RecA nucleation, SsbA, SsbB

## Abstract

Natural plasmid transformation plays an important role in the dissemination of antibiotic resistance genes in bacteria. During this process, *Bacillus subtilis* RecA physically interacts with RecU, RecX, and DprA. These three proteins are required for plasmid transformation, but RecA is not. *In vitro*, DprA recruits RecA onto SsbA-coated single-stranded (ss) DNA, whereas RecX inhibits RecA filament formation, leading to net filament disassembly. We show that a null *recA* (Δ*recA*) mutation suppresses the plasmid transformation defect of competent Δ*recU* cells, and that RecU is essential for both chromosomal and plasmid transformation in the Δ*recX* context. RecU inhibits RecA filament growth and facilitates RecA disassembly from preformed filaments. Increasing SsbA concentrations additively contributes to RecU-mediated inhibition of RecA filament extension. DprA is necessary and sufficient to counteract the negative effect of both RecU and SsbA on RecA filament growth onto ssDNA. DprA-SsbA activates RecA to catalyze DNA strand exchange in the presence of RecU, but this effect was not observed if RecU was added prior to RecA. We propose that DprA contributes to RecA filament growth onto any internalized SsbA-coated ssDNA. When the ssDNA is homologous to the recipient, DprA antagonizes the inhibitory effect of RecU on RecA filament growth and helps RecA to catalyze chromosomal transformation. On the contrary, RecU promotes RecA filament disassembly from a heterologous (plasmid) ssDNA, overcoming an unsuccessful homology search and favoring plasmid transformation. The DprA–DprA interaction may promote strand annealing upon binding to the complementary plasmid strands and facilitating thereby plasmid transformation rather than through a mediation of RecA filament growth.

## Introduction

Natural transformation, described for first time some 90 years ago (Griffith, 1928), is an important horizontal gene transfer mechanism for the spread of metabolic pathways and antibiotic resistance genes, as well as for the emergence of infections and opportunistic pathogens (Doolittle, 1999; Fraser et al., 2007; Takeuchi et al., 2014). Development of natural competence allows for efficient uptake of any environmental double-stranded (ds) DNA, and its internalization into the cytosol as linear single-stranded (ss) DNA (Chen and Dubnau, 2004; Claverys et al., 2009; Kidane et al., 2012). If the incoming DNA shares homology with the host DNA, the ssDNA can be integrated into the recipient genome via RecA-mediated homologous recombination (chromosomal transformation). When there is no homology with the recipient, and the incoming ssDNA has a replicon and internal homology (e.g., an oligomeric plasmid molecule), a circular replicon is reconstituted in a RecA-independent manner (plasmid transformation) (Claverys et al., 2009; Kidane et al., 2012). In the absence of DNA homology with the recipient and with itself, the internalized linear ssDNA is degraded (Chen and Dubnau, 2004; Claverys et al., 2009; Kidane et al., 2012).

Identification of the factors that contribute to the transfer of antibiotic resistance genes, mainly by plasmid-borne genes, is an important unanswered problem in evolutionary biology. Furthermore, the human and economic cost of increased antibiotic resistance is enormous, and basic information is needed to intercept this spread. To understand the molecular basis of natural plasmid transformation, we have used competent *Bacillus subtilis* cells as a model. In this bacterium, transient natural competence is induced in a subset of cells by starving them of critical nutrients (Chen and Dubnau, 2004; Kidane et al., 2012). In the competent subpopulation, DNA replication is halted, expression of a set of genes is induced (including *recA, ssbA* and competence-specific *dprA* and *ssbB*), and the DNA uptake apparatus is built at one cell pole (Chen and Dubnau, 2004; Kidane et al., 2012). This apparatus binds environmental dsDNA, degrades one of the strands, and internalizes the other strand independently of its nucleotide sequence and polarity (Chen and Dubnau, 2004; Kidane et al., 2012; Takeuchi et al., 2014). Cytosolic RecA transiently localizes to the cell pole, and co-localizes with SsbA, SsbB, DprA, RecU and proteins of the DNA uptake apparatus (Hahn et al., 2005; Kramer et al., 2007; Tadesse and Graumann, 2007; Kidane et al., 2009). When plasmid or chromosomal dsDNA is added, RecA forms threads emanating from the ssDNA entry site toward the nucleoid, and RecX co-localizes with the RecA threads (Kidane et al., 2009; Cárdenas et al., 2012).

**FIGURE 1:**
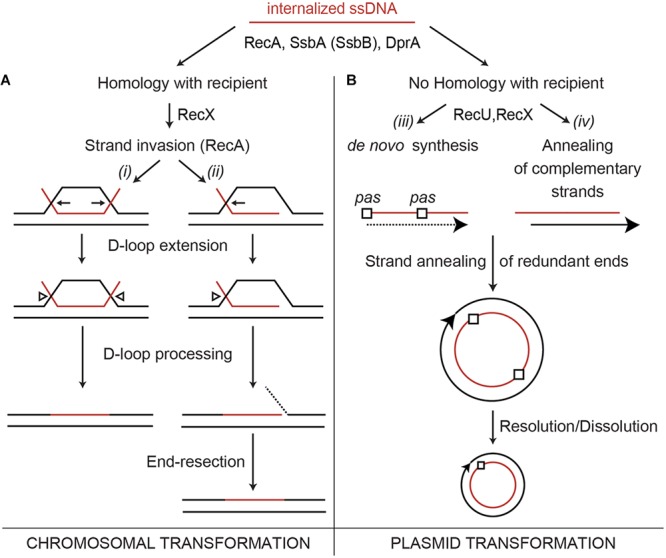
Chromosome and plasmid transformation. The DNA uptake apparatus, built at one of the cell poles, internalizes one of the strands as linear ssDNA. **(A)** During chromosomal transformation DprA (or RecO in the Δ*dprA* context *in vivo*) loads RecA onto SsbA (or SsbA and SsbB)-coated ssDNA. During the homology search, the RecA filament length is modulated by RecX. **(Ai,ii)**, a RecA nucleoprotein filament catalyzes DNA strand invasion. Then, the D-loops are extended (denoted by pointing arrows), and an uncharacterized resolvase cleaves the displaced strand (arrow head), which is then degraded. **(B)** RecA filaments form on the internalized oligomeric plasmid ssDNA. RecA inhibition by RecU or RecX facilitates the conversion of the ssDNA into its duplex form via DNA synthesis (initiated at the primosome assembly site, *pas*) to generate a strand with the opposite polarity (broken line) **(Biii)** or via annealing of the two independently internalized complementary strands **(Biv)**. Then, the annealing of the terminal redundant DNA ends allows circularization of the oligomeric plasmid DNA **(Biii,iv)**. In a final step, the oligomeric plasmid molecule is converted into a monomer.

The different accessory factors that assist RecA during chromosomal transformation can be divided into two broad classes, those that act before (mediators) and those that act during the homology search and the DNA strand exchange reaction (modulators) (Beernink and Morrical, 1999). Some of these factors are specific for natural transformation (e.g., DprA and SsbB) or recombinational repair (RecR and RecF), and some participate in both processes (RecX and RecU) (**Figure 1**; Kidane et al., 2012). The mediators that participate in natural transformation are further divided into those that promote (DprA and RecO), limit (SsbA and SsbB), or activate RecA to catalyze DNA strand exchange in the presence of ATP (SsbA together with DprA or RecO) (Yadav et al., 2014; Carrasco et al., 2015, 2016). Modulators are divided into those that promote RecA nucleoprotein filament assembly (RecF) or disassembly (RecX) (Carrasco et al., 2005; Cárdenas et al., 2012; Le et al., 2017).

RecA from the phylum Firmicutes (*B. subtilis, Streptococcus pneumoniae*) nucleates and polymerizes onto naked ssDNA in the ATP bound form (RecA⋅ATP), but these RecA nucleoprotein filaments cannot catalyze DNA strand exchange (Lovett and Roberts, 1985; Steffen et al., 2002; Cañas et al., 2008; Yadav et al., 2014), which suggests that the main role of mediators is to recruit and activate RecA⋅ATP (Yadav et al., 2014). The essential SsbA [counterpart of *Escherichia coli* SSB (SSB*_Eco_*)], binds ssDNA with >fivefold higher affinity than SsbB, and with >500-fold higher affinity than DprA or RecA (Yadav et al., 2012, 2014). During the transient stage of natural competence, SsbA might bind to the internalized ssDNA as soon as it leaves the entry channel of the DNA uptake apparatus. SsbA competes with RecA⋅ATP for nucleation sites on ssDNA (Cox, 2007; Bell and Kowalczykowski, 2016). RecA⋅ATP cannot remove SsbA when RecA extension from another nucleation site reaches an SsbA-binding site, and the presence of DprA is necessary to reverse the negative effect of SsbA (Yadav et al., 2014). Following interaction with the SsbA protein, DprA generates a DNA structure competent for RecA loading and activation. This activation is facilitated by a DprA–RecA interaction (Mortier-Barriere et al., 2007; Mirouze et al., 2013; Lisboa et al., 2014). A RecA nucleoprotein filament binds transiently and non-sequence-specifically to multiple regions of the centrally located chromosome, to search efficiently for a unique homologous sequence (Bell and Kowalczykowski, 2016). When homology is found, the RecA nucleoprotein filament initiates strand invasion by forming a nascent three-stranded synaptic-joint [displaced loop (D-loop)] (**Figure 1Ai,ii**). With the help of mediators and modulators, RecA⋅ATP catalyzes DNA strand exchange of the incoming linear ssDNA with the recipient non-replicating haploid genome (**Figure 1A**).

Naturally occurring plasmids share little or no DNA homology with the recipient bacterium, thus plasmid transformation follows a different mechanism than chromosomal transformation. It was early proposed that a single oligomeric plasmid molecule is established 1,000-fold more effective than a monomeric molecule via a RecA-independent mechanism (Canosi et al., 1978). Two current models, requiring a different degree of DNA strand annealing, account for plasmid establishment (**Figure 1Biii,iv**). In the first model, the replication machinery assembles at one of the primosome assembly sites (*pas*) or at the lagging strand replication origin (in rolling circle replicating plasmids) of an oligomeric plasmid ssDNA to initiate the synthesis of the complementary strand. Annealing of the complementary ssDNA ends results in the circularization, and subsequent ligation (**Figure 1Biii**; Lacks, 1988). The second model proposes that the complementary oligomeric strands enter, through the DNA uptake machinery, into the same bacterium. The annealing of these complementary strands results in complementary ssDNA ends, circularization and subsequent ligation of the plasmid molecule (**Figure 1Biv**). Then, the established oligomeric plasmid is replicated and resolved or dissolved leading to a plasmid monomer during exponential growth (**Figure 1B**; Kidane et al., 2012). We favor the second model because previous experiments show that: (i) less than 300-bases of newly synthesized DNA are present in an average recombinant plasmid molecule during the transient stage of natural competence (Weinrauch and Dubnau, 1987); (ii) plasmid transformation has two-hit kinetics; and (iii) plasmids with or without a *pas* site or lagging strand origin of replication transform competent cells with a similar efficiency (Kidane et al., 2012).

Genetic analysis showed that a lack of RecA blocks chromosomal transformation (>10^4^-fold), but it does not affect transformation of naturally occurring oligomeric plasmids in otherwise wild type (*rec****^+^***) competent cells (Canosi et al., 1978; Alonso et al., 1991). Inactivation of RecX decreases both chromosomal and plasmid transformation by ∼200- and ∼60-fold, respectively, and the absence of DprA also reduces the chromosomal and plasmid transformation by ∼75- and ∼45-fold, respectively (Cárdenas et al., 2012; Le et al., 2017). A lack of RecU reduces plasmid transformation by ∼35-fold (Cañas et al., 2008), and the absence of RecF marginally impairs (<twofold) both chromosomal and plasmid transformation (Alonso et al., 1991). Recently, it was shown that the RecA-mediated search for homology is unproductive and wasteful when the incoming ssDNA shares no homology with the recipient. After a search for homology, the RecA filaments are disassembled by RecX. This anti-recombination effect of RecX was found to be regulated by the DprA and SsbA proteins, although both proteins might not interact physically with RecX (Le et al., 2017). RecA inhibition by RecX is required for plasmid transformation and reversing this, by DprA and SsbA, would favor RecA-mediated homology search and chromosomal transformation (Le et al., 2017).

The contribution of RecU to plasmid transformation is poorly understood. RecU, which is present in bacteria of the phylum Firmicutes, Archaea and some viruses, is a genuine Holliday junction (HJ)-resolving enzyme, structurally unrelated to the ubiquitous RuvC*_Eco_* HJ resolvase (Ayora et al., 2004; McGregor et al., 2005). RecU has at least two activities: to cleave HJ recombination intermediates in concert with the RuvAB branch migration translocase, and to contribute to plasmid transformation. The structure of RecU has a mushroom-like appearance, with a cap and a stalk region (not present in other enzymes of the family) (McGregor et al., 2005; Khavnekar et al., 2017). Biochemical assays show that the stalk region of RecU, which penetrates the center of the HJ to distort it, interacts physically with RecA even in the apo form (Carrasco et al., 2005, 2009; Cañas et al., 2008). *In vitro*, RecU negatively modulates RecA⋅dATP activities, although dATP is not the physiological nucleotide cofactor used by RecA (Yadav et al., 2014). If the biological role of RecU during natural plasmid transformation is the regulation of RecA⋅ATP nucleoprotein filament formation remains elusive.

To determine whether RecU regulates RecA⋅ATP nucleation and to elucidate the molecular mechanisms that lead RecU to act as a RecA modulator during plasmid transformation, we studied RecA⋅ATP nucleation and filament extension onto ssDNA in the presence of RecU. We show that RecU inhibited RecA⋅ATP nucleation and polymerization onto ssDNA *in vitro*, and that this effect was additive when it was combined with SsbA or SsbB. RecU promoted depolymerization of preformed RecA filaments. DprA reversed the negative effect of RecU or SsbA on RecA filament extension and on RecA-mediated strand exchange. We speculate that DprA-mediated RecA nucleation and filament growth and DprA-mediated strand annealing might be mutually exclusive. The positive contribution of RecU to plasmid transformation is to counteract the deleterious effects of RecA filaments on heterologous ssDNA. We show that the plasmid transformation defect of Δ*recU* (Δ*recX*) cells is overcome by mutating RecA. We propose that RecU might limit RecA filament formation and favors DprA-mediated strand annealing of SsbA- (or SsbB)-coated complementary plasmid strands to facilitate plasmid transformation.

## Results

### RecU Contributes to Plasmid Transformation in *rec^+^* Cells and Also to Chromosomal Transformation in the *ΔrecX* Context

The defects of single Δ*recA*, Δ*dprA*, Δ*recX*, and Δ*recU* mutant strains in chromosomal and plasmid transformation have been analyzed (Alonso et al., 1988; Tadesse and Graumann, 2007; Cañas et al., 2008; Kidane et al., 2009; Cárdenas et al., 2012; Yadav et al., 2013; Le et al., 2017). Here we re-evaluated these strains for direct comparison. A lack of DprA or RecX reduced both chromosomal and plasmid transformation, the absence of RecA blocked chromosomal transformation, and the absence of RecU reduced the plasmid transformation frequency of haploid non-replicating competent cells (**Table 1**).

**Table 1:** The role of RecU in plasmid transformation is superseded in the *recA* context.

Strain^a^	Relevant genotype	Normalized chromosomal transformation	Normalized plasmid transformation
BG214	*rec****^+^***	100 (4.1 × 10^5^)	100 (9.1 × 10^3^)
BG190	Δ*recA*	<0.01	98 ± 2
BG1163	Δ*dprA*	1.3 ± 0.4	2.2 ± 0.5
BG855	Δ*recX*	0.46 ± 0.2	1.6 ± 0.2
BG855	Δ*recU*	51 ± 2.0	2.6 ± 0.6
BG703	Δ*ruvAB*	78 ± 7	37 ± 5
BG651	Δ*recU* Δ*recA*	<0.01	44 ± 3
BG1147	Δ*recX* Δ*recA*	<0.01	55 ± 8
BG1081	Δ*recU* Δ*recX*	<0.01	<0.01
BG1609	Δ*recU* Δ*dprA*	0.8 ± 0.4	0.9 ± 0.3
BG1291	Δ*dprA* Δ*recA*	<0.01	0.4 ± 0.1

*^*a*^All strains are isogenic with BG214 (rec***^+^***). The genotype of the parental strain is trpCE metA5 amyE1 ytsJ1 rsbV37 xre1 xkdA1 att^*SP*β^ att^*ICEBs1*^. Competent B. subtilis cells auxotrophic for methionine were transformed with 0.1 μg of chromosomal DNA from the prototroph SB19 strain to met****^+^****or with the pUB110 plasmid DNA to Nm^*R*^. The yield of met****^+^****(chromosomal transformation) and Nm^*R*^ transformants (plasmid pUB110 transformation) was corrected for DNA uptake and cell viability, and the values obtained were normalized relative to that of the rec****^+^****strain, recorded as 100 (in parentheses, number of transformants per 0.1 μg DNA). Results are shown as mean of at least seven independent experiments.*

At least two types of mechanisms for RecU activity during competence can be envisioned. (i) RecU, in concert with the branch migration translocase RuvAB, might process D-loops that could form during natural chromosomal transformation (see **Figure 1A**); and (ii) RecU may regulate RecA activities to facilitate plasmid transformation (see **Figure 1B**). If the first hypothesis is correct, the lack of RuvAB would also impair plasmid transformation. The absence of RuvAB is synthetically lethal in the Δ*recU* context (Sanchez et al., 2005), thus only single mutants can be tested here. The absence of RuvAB marginally reduced both chromosomal (1.3-fold) and plasmid transformation (2.7-fold) in otherwise competent *rec****^+^*** cells (**Table 1**). Similarly, a lack of prophage SKIN-encoded RusA-like HJ resolvase does not impair natural transformation (Kidane et al., 2012). If the second assumption is correct, a lack of RecA would overcome the need for RecU in plasmid transformation. The absence of RecA partially suppressed the RecU defect in plasmid transformation, but chromosomal transformation was abolished (**Table 1**), as reported for cells lacking RecA (Alonso et al., 1988). Similarly, a lack of RecA superseded the need for RecX during plasmid transformation (**Table 1**; Le et al., 2017). These data suggest that: (i) RecA filament formation on incoming homologous ssDNA is essential for chromosomal transformation, but filament formation on heterologous plasmid ssDNA might be deleterious in otherwise competent Δ*recU* (or Δ*recX*) cells; and (ii) RecA inhibition by RecU (or RecX) is required for plasmid transformation in otherwise *rec****^+^*** cells.

To study whether RecX and RecU contribute independently to plasmid transformation, we constructed the Δ*recX* Δ*recU* strain. Chromosomal and plasmid transformation was reduced by >10^4^-fold in competent Δ*recX* Δ*recU* cells (**Table 1**). These results suggest that: (i) there is a division of labor between RecX and RecU modulators, but a certain degree of redundancy might mask the role of RecU in controlling RecA activities during chromosomal transformation; (ii) RecU, in the absence of RecX, might work as the main RecA modulator contributing to RecA-mediated chromosomal transformation; and (iii) RecU and RecX additionally contribute to overcome the negative effect of the unproductive RecA filaments on the heterologous ssDNA as judged by the plasmid transformation efficiency in Δ*recA* vs. *rec****^+^*** cells (**Table 1**).

*In vitro* DprA has at least two activities: to recruit RecA onto SsbA-coated ssDNA and to mediate annealing between two complementary SsbA-coated DNA strands (Yadav et al., 2013). To test whether DprA works in concert with RecU during plasmid transformation, we constructed the Δ*dprA* Δ*recU* strain. The absence of DprA and RecU reduced the amount of chromosomal and plasmid transformants, as did the competent Δ*dprA* cells (**Table 1**). Similarly, RecX is epistatic to DprA during chromosomal and plasmid transformation (Le et al., 2017). Inactivation of RecA and DprA blocked chromosomal transformation and reduced plasmid transformation as the most affected single mutant strains. In summary, RecU is epistatic to DprA, but not to RecX during plasmid transformation; and a lack of RecA supersedes the need for RecU (and RecX), but not of DprA in plasmid transformation (**Table 1**). In other words, reversing RecA inhibition by RecU (or RecX) would favor RecA-mediated homology search and chromosomal transformation, but in the absence of DNA homology DprA-mediated DNA strand annealing and plasmid transformation would be favored.

### RecU and SsbA Inhibit the ATPase Activity of RecA

To determine how RecU may regulate RecA during plasmid transformation, we evaluated the kinetics of ssDNA-dependent ATP hydrolysis by RecA as an indirect readout of RecA nucleation and polymerization onto naked or SSB-coated ssDNA. The lag phase to reach the maximal rate of ATP hydrolysis (or time of nucleation) in this reaction permits evaluation of the nucleation step and the *K*_cat_ of the ATP hydrolysis gives clues about how modulators act at the RecA polymerization step (Cox, 2007; Bell and Kowalczykowski, 2016).

**FIGURE 2:**
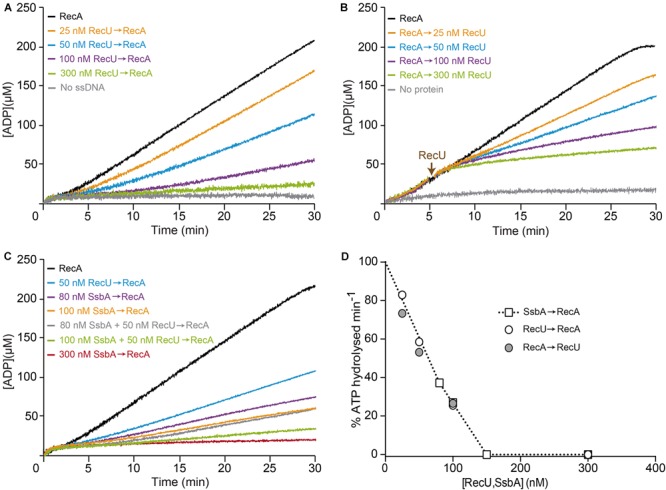
Effect of RecU on RecA nucleation on ssDNA and filament growth. **(A)** Circular 3,199-nt ssDNA (10 μM) was preincubated with increasing RecU concentrations (5 min, 37°C) in buffer B containing 5 mM ATP. RecA was added and ATPase activity was measured (30 min, 37°C). **(B)** ssDNA was preincubated with RecA (0.8 μM; 5 min, 37°C), followed by increasing RecU concentrations, and ATPase activity was measured. **(C)** ssDNA was preincubated with increasing SsbA concentrations and/or a fixed amount of RecU (5 min, 37°C), followed by RecA, and ATPase activity was measured. The amount of ATP hydrolyzed was calculated as described (see Materials and Methods). The + symbol indicates that proteins were preincubated, and the arrow indicates the order of protein addition. Representative graphics are shown here, and quantification of the results is expressed as the mean ± SEM of >3 independent experiments (see **Table 2**). **(D)** Comparison of the inhibitory effect of RecU and SsbA in RecA filament formation using the kinetic assays of *A*-*C* (see **Table 2**).

The physiological concentrations of monomeric RecA and dimeric RecU in exponentially growing cells are ∼34 (basal level 5.5 μM) and ∼2.5 μM, respectively (Carrasco et al., 2009; Cárdenas et al., 2014). RecA-mediated ATP hydrolysis increased without an apparent lag phase and catalytic constant (*K*_cat_) values near the previously observed *K*_cat_ of 9.4 ± 0.4 min^-1^ (1 RecA monomer/12-nt, 800 nM or ∼40-fold below physiological concentrations) (**Figure 2** and **Table 2**; Steffen and Bryant, 1999; Yadav et al., 2014). When ssDNA was omitted, RecA-mediated ATP hydrolysis was not observed (**Figure 2A**).

**Table 2:** Rates of ssDNA-dependent ATP hydrolysis and lag time measurements.

Proteins^a^	Lag time^a^ (min)	*K*_cat_ min^-1a^
RecA 800 nM (1 RecA/12-nt)^b^	<1	9.4 ± 0.5
RecU/400-nt → RecA	∼4	7.8 ± 0.3
RecA → RecU/400-nt	<1	6.9 ± 0.2
RecU/200-nt → RecA	∼4	5.5 ± 0.2
RecA → RecU/200-nt	ND	5.0 ± 0.3
RecU/100-nt → RecA	∼4	2.4 ± 0.2
RecA → RecU/100-nt	–	2.5 ± 0.3
RecU/33-nt → RecA	–	<1
RecA → RecU/33-nt	–	<1
SsbA/125-nt → RecA	<1	3.5 ± 0.3
SsbA/100-nt → RecA	<1	2.5 ± 0.3
SsbA/33-nt → RecA	–	<1
SsbA/125-nt + RecU/200-nt → RecA	<1	2.5 ± 0.2
SsbA/100-nt + RecU/200-nt → RecA	–	<1
RecA → SsbA/33-nt + RecU/200-nt	–	<1
RecA → SsbB/33-nt + RecU/200-nt	–	<1

RecA^b^	<1	9.0 ± 0.4
RecU/100-nt → RecA	<1	2.5 ± 0.3
DprA/1,600-nt → RecA	<1	10.1 ± 0.2
DprA/1,600-nt + RecU/100-nt → RecA	<1	3.8 ± 0.3
DprA/1,600-nt + RecA → RecU/100-nt	<1	4.8 ± 0.4
DprA/800-nt → RecA	<1	12.5 ± 0.3
DprA/800-nt + RecU/100-nt → RecA	<1	6.0 ± 0.4
DprA/800-nt + RecA → RecU/100-nt	<1	5.5 ± 0.3
DprA/400-nt → RecA	<1	12.5 ± 0.2
DprA/400-nt + RecU/100-nt → RecA	<1	9.0 ± 0.2
DprA/400-nt + RecA → RecU/100-nt	<1	7.3 ± 0.3
DprA/100-nt → RecA^b^	<1	12.5 ± 0.2
DprA/100-nt + RecU/100-nt → RecA	<1	12.0 ± 0.4
DprA/100-nt + SsbA/33-nt → RecA^b^	<1	15.0 ± 0.2
SsbA/33-nt + RecU/100-nt → RecA	<1	<1
SsbA/33-nt + RecU/100-nt → DprA/100-nt + RecA	<1	12.5 ± 0.4
DprA/100-nt + SsbB/33-nt → RecA^b^	<1	14.3 ± 0.2
SsbB/33-nt + RecU/100-nt → RecA	<1	<1
SsbB/33-nt + RecU/100-nt → DprA/100-nt + RecA	<1	13.5 ± 0.2

*^*a*^Rates of RecA-mediated ATP hydrolysis and nucleation lag times were measured as indicated (see Materials and Methods). ^*b*^RecA-mediated ATP hydrolysis was reported elsewhere (Yadav et al., 2014; Carrasco et al., 2015), and determined here for direct comparison. The kinetic parameters for RecA were derived from more than three independent experiments like those shown in **Figures 2, 6, 7**; results are presented as mean* ± *SEM. Concentrations of all other components are listed individually. The mean ATP hydrolysis rate was obtained from more than three independent experiments. –, not done.*

Limiting RecU concentrations (25–50 nM RecU, 1 RecU dimer/400 and 200-nt, or 100- and 50-fold below physiological concentrations) delayed RecA nucleation (lag phase of ∼4 min) and reduced the rate of RecA-catalyzed ATP hydrolysis (*K*_cat_ ∼7.8 and ∼5.5 min^-1^, respectively) (**Figure 2A** and **Table 2**). At sub-saturating RecU concentrations (100 nM RecU, or 1 RecU dimer/100-nt) and with a low RecU:RecA molar ratio (1:8), RecA-mediated ATP hydrolysis was inhibited (*K*_cat_ ∼2.4 min^-1^), suggesting that each RecU dimer does not have to interact with every RecA monomer on the filament to exert its inhibitory effect. Stoichiometric RecU concentrations with respect to ssDNA (1 RecU/33-nt, which corresponds to a RecU:RecA molar ratio of 1:2.6) blocked RecA-mediated ATP hydrolysis (*K*_cat_ < 1 min^-1^) (**Figure 2A** and **Table 2**).

To test whether RecU could impede RecA filament growth by competing for ssDNA binding and/or interacting with RecA, preformed RecA filaments were incubated with increasing amounts of RecU, and RecA-mediated ATP hydrolysis was measured. Addition of increasing RecU concentrations (1 RecU/400- to 33-nt) reduced, inhibited and blocked RecA-mediated ATP hydrolysis (*K*_cat_ ∼6.9, 5.0, 2.5 and <1 min^-1^), respectively (**Figure 2B** and **Table 2**).

At physiological pH, SsbA binds ssDNA [apparent binding constants (*K*_D_) < 0.2 nM] with higher affinity than RecU (*K*_D_ ∼200 nM) or RecA⋅ATPγS (*K*_D_ > 500 nM) (Yadav et al., 2012). One SsbA (or SsbB) tetramer occludes 65- or 35-nt (Shereda et al., 2008), and one RecU dimer binds ∼35-nt (Carrasco et al., 2005). RecA⋅ATP binds ssDNA at a stoichiometry of 3-nt/monomer to form a right-handed nucleoprotein filament (Chen et al., 2008), also termed pre-synaptic filament. The physiological concentrations of tetrameric SsbA in exponentially growing cells is ∼1.3 μM (Carrasco et al., 2009; Cárdenas et al., 2014). Stoichiometric SsbA concentrations (1 SsbA tetramer/33-nt, 300 nM) block RecA-catalyzed ATP hydrolysis (**Figure 2C**; Yadav et al., 2014). To test whether SsbA can compete with RecU to inhibit RecA assembly on ssDNA, limiting SsbA (80–100 nM, 1 SsbA/125- to 100-nt, or 16- and 13-fold below physiological concentrations) and RecU (1 RecU/200-nt) concentrations were preincubated with ssDNA, followed by the addition of RecA⋅ATP. Under this condition, RecA-catalyzed ATP hydrolysis was inhibited and blocked in the presence of fixed RecU and increasing SsbA concentrations, respectively (**Figure 2C** and **Table 2**), suggesting an additive inhibitory effect of both RecU and SsbA proteins on RecA nucleation. We found a linear relationship between RecU or SsbA concentration and inhibition of RecA-mediated ATP hydrolysis, which is consistent with a non-catalytic mechanism for the inhibition of RecA ATPase activity (**Figure 2D**). We suggest that RecA⋅ATP passively nucleates on free ssDNA segment not bound by RecU and/or SsbA proteins.

### Three-Way Junction DNA Poorly Competes for RecU-Mediated Inhibition of RecA ATPase Activity

**FIGURE 3:**
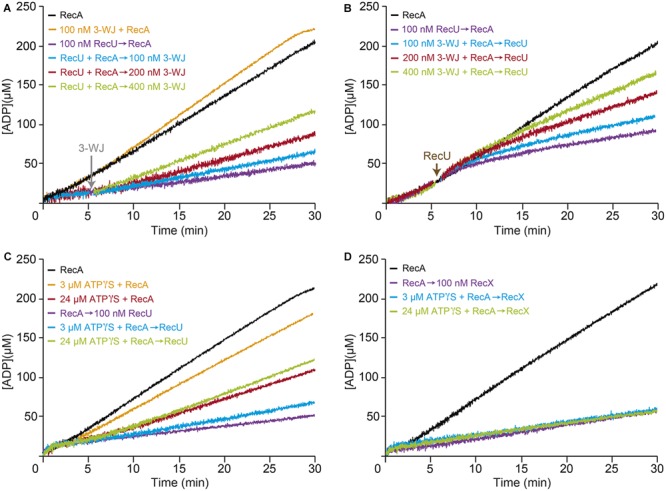
Effect of RecU on RecA nucleation on ssDNA and filament growth in the presence of 3-WJ DNA. Circular 3,199-nt ssDNA (10 μM in nt) was preincubated with RecA (0.8 μM) and RecU (100 nM) **(A)**, or with RecA (0.8 μM) and increasing concentrations of 3-WJ DNA (100–400 nM in DNA molecules) **(B)** (5 min, 37°C) in buffer B containing 5 mM ATP. Increasing concentrations of 3-WJ DNA **(A)** or a fixed amount of RecU **(B)** was then added and ATPase activity was measured (30 min, 37°C). **(C,D)** The inhibitory effect of RecU on RecA filament extension is different than the one exerted by RecX. Circular ssDNA (10 μM) was preincubated with a fixed amount of RecA (0.8 μM) (5 min, 37°C) in buffer B containing limiting ATPγS (3 or 24 μM). A fixed amount of RecU **(C)** or RecX **(D)** (100 nM) and 5 mM ATP were added, and ATPase activity was measured. As controls, circular ssDNA (10 μM) was preincubated with RecA in buffer B lacking or containing limiting ATPγS (3 or 24 μM), then ATP was added and ATPase activity was measured. The amount of ATP hydrolyzed was calculated as described. Representative graphics are shown here and quantification of the results is expressed as the mean ± SEM of >3 independent experiments [see Supplementary Table S1, for parts **(A,B)**, and Supplementary Table S2, for parts **(C,D)**].

RecU binds to D-loop or three-way junction (3-WJ) DNA with ∼180-fold higher affinity than ssDNA with a half-life longer than 20 min (Ayora et al., 2004; Cañas et al., 2011), suggesting that RecU, at equimolar concentrations with 3-WJ DNA should primarily be in a protein-DNA complex rather than bound to ssDNA. The results from the previous section suggest that RecU inhibits RecA-mediated ATP hydrolysis, perhaps by competing with RecA for ssDNA binding (**Figure 2D**). Alternatively, if the inhibitory effect is indirect, RecU bound to ssDNA might interact with another discrete ssDNA region to promote formation of DNA secondary structures to which RecA cannot bind. To test these hypotheses, we measured the kinetics of RecA-mediated ATP hydrolysis in the presence of ssDNA and increasing concentrations of 3-WJ competitor DNA. The 3-WJ DNA did not reduce the amount of RecA-mediated ATP hydrolysis (**Figure 3A** and Supplementary Table S1).

Limiting RecU concentrations (1 RecU/100-nt) inhibited RecA-mediated ATP hydrolysis (*K*_cat_ ∼2.4 min^-1^) (**Figure 3A** and Supplementary Table S1). Addition of 3-WJ DNA at 1:1 or 1:2 molar RecU:3-WJ DNA ratios did not reverse the negative effect of RecU on RecA nucleation or filament extension onto ssDNA (*K*_cat_ ∼2.9 and ∼3.5 min^-1^, respectively). An excess of 3-WJ DNA (1:4 molar RecU:3-WJ DNA ratio) was needed to partially reverse the negative effect of RecU in RecA-mediated ATP hydrolysis (*K*_cat_ ∼5.4 min^-1^) (**Figure 3A** and Supplementary Table S1). Then, RecA was allowed to nucleate onto ssDNA in the presence of increasing 3-WJ DNA concentrations. RecU addition, at a 1:1 RecU:3-WJ DNA ratio, did not reverse its inhibitory effect in RecA-mediated ATP hydrolysis (*K*_cat_ ∼2.7 min^-1^) compared to in the absence of the competitor (*K*_cat_ ∼2.5 min^-1^) (**Figure 3B** and Supplementary Table S1). Under this reaction condition, RecU is presumed to be bound to its high affinity substrate, the 3-WJ DNA. It is likely that, independent of the order of addition, RecU bound to a D-loop or 3-WJ DNA at 1:2 or 1:4 molar RecU:3-WJ DNA ratios still inhibits RecA-mediated ATP hydrolysis (*K*_cat_ ∼4.0 and ∼6.3 min^-1^), respectively (**Figure 3B** and Supplementary Table S1).

### RecU and RecX Inhibit RecA-Mediated ATP Hydrolysis by Distinct Mechanisms

The RecU and RecX modulators interact physically with RecA (Carrasco et al., 2005; Cañas et al., 2008; Le et al., 2017). RecX actively disassembles RecA from the ssDNA (Le et al., 2017). To test whether RecU uses a similar mechanism to inhibit RecA, it was preincubated with ssDNA with limiting concentrations of the slowly hydrolysable ATP analog ATPγS (3 and 24 μM). After addition of 5 mM ATP, RecA (800 nM) preincubated with 3 μM ATPγS (below *K*_m_) did not significantly affect its ATPase activity (*K*_cat_ of ∼8.9 min^-1^), however, with 24 μM ATPγS (at about *K*_m_), RecA-mediated ATP hydrolysis (*K*_cat_ of ∼4.8 min^-1^) was reduced when compared to the absence of ATPγS (*K*_cat_ of ∼9.4 min^-1^) (**Figure 3C** and Supplementary Table S2).

When a limiting RecU concentration (100 nM) and 5 mM ATP were added to the preformed ssDNA-RecA⋅ATPγS complexes (time 0), the inhibitory effect of RecU was marginally affected in the presence of 3 μM ATPγS. RecA filament assembly in the presence of ATPγS, however, led to partial reversal of the negative effect of RecU on RecA-mediated ATP hydrolysis (*K*_cat_ of ∼5.2 min^-1^) (**Figure 3C** and Supplementary Table S2). It is likely that the small fraction of RecA⋅ATPγS bound to ssDNA might contribute to stabilization of RecA on the ssDNA, and the increased stability of the RecA filament is able to partially bypass the inhibitory effect of RecU on RecA-mediated ATP hydrolysis. Alternatively, RecU might inhibit RecA nucleation onto ssDNA.

In the absence of ATPγS, limiting RecX (100 nM) concentrations inhibited ATP hydrolysis to a similar extent to that of limiting RecU concentrations (**Figure 3D** and Supplementary Table S2). However, RecA assembled on the ssDNA at limiting ATPγS (3 and 24 μM) concentrations was unable to overcome the negative effect of RecX (**Figure 3D** and Supplementary Table S2). The mechanism used by RecU to inhibit RecA-mediated ATP hydrolysis thus appears to differ from the active RecX*_Eco_*/RecX capping mechanism (see Ragone et al., 2008; Le et al., 2017).

### RecU and SSB Additively Inhibit RecA⋅dATP Nucleation on a RecU-ssDNA-SSB Complex

The data presented in **Figure 3C** suggests that RecU is less effective at inhibiting RecA-mediated ATP hydrolysis if the RecA-ssDNA complexes are stabilized by ATPγS. *Escherichia coli* and *B. subtilis* RecA, in the dATP-bound form (RecA⋅dATP), adopts an active state with a greater affinity for ssDNA and a more highly cooperative polymerization onto ssDNA than RecA⋅ATP (Lovett and Roberts, 1985; Kowalczykowski et al., 1987; Manfredi et al., 2008).

**FIGURE 4:**
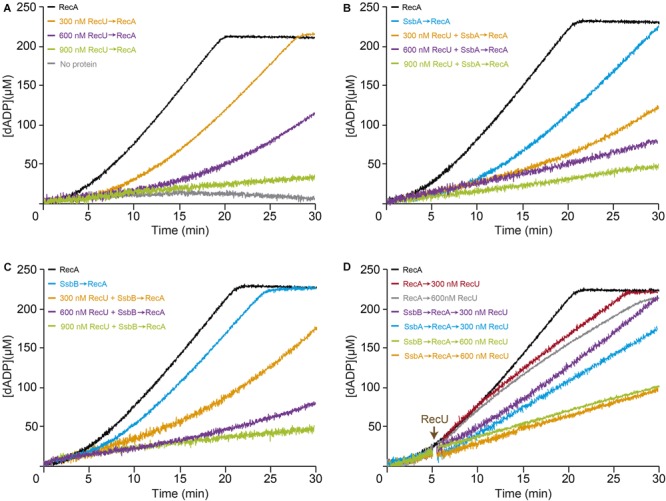
Effect of RecU on RecA nucleation on ssDNA and filament growth in the presence of dATP. Circular 3,199-nt ssDNA (10 μM in nt) was preincubated with increasing RecU concentrations **(A)** and a fixed amount of SsbA **(B)** or SsbB **(C)** (300 nM) (5 min, 37°C) in buffer B containing 5 mM dATP. RecA (0.8 μM) was added and dATPase activity was measured (30 min, 37°C). **(D)** ssDNA was preincubated with a fixed concentration of RecA and SsbA or SsbB (5 min, 37°C), followed by increasing RecU concentrations, and dATPase activity was measured. The amount of dATP hydrolyzed was calculated as described. Representative graphics are shown here and quantification of the results is expressed as the mean ± SEM of >3 independent experiments (see Supplementary Table S3).

To address whether stabilization of a RecA-ssDNA filament by dATP counteracts the negative effect of RecU on RecA nucleoprotein filament formation, RecA-mediated dATP hydrolysis was measured in the presence of RecU. As observed previously, limiting RecA⋅dATP (1 RecA/12-nt, 800 nM) concentrations resulted in curves with a ∼4 min lag phase followed by robust dATP hydrolysis, with a maximal rate of ∼17.8 min^-1^ (**Figures 4A–D** and Supplementary Table S3; Lovett and Roberts, 1985; Steffen and Bryant, 1999; Yadav et al., 2012). Addition of stoichiometric RecU concentrations (300 nM) slightly reduced the maximal dATP hydrolysis rate (1.4-fold), but the RecA nucleation time was significantly delayed (∼9 min lag phase) (**Figure 4A** and Supplementary Table S3). Saturating RecU concentrations (1 RecU/16-nt) delayed (∼13 min lag) and reduced RecA nucleation (*K*_cat_ 10.2 min^-1^) (**Figure 4A** and Supplementary Table S3). Equimolar RecU:RecA ratios (1:1) (1 RecU/11-nt, 900 nM) were necessary to block RecA-catalyzed dATP hydrolysis (*K*_cat_ < 1 min^-1^) (**Figure 4A** and Supplementary Table S3; Carrasco et al., 2005; Cañas et al., 2008). RecU inhibition of RecA-mediated ATP hydrolysis is due to the presence of RecU, rather than an indirect effect (**Figure 2A** vs. **Figure 4A**). Indeed, a RecU division of labor mutant [Tyr80-to-Ala substitution (Y80A)], which cleaves a HJ, but does not interact with RecA, fails to inhibit the dATPase activity of RecA (Cañas et al., 2011). It is likely that by increasing the affinity of RecA for ssDNA (in the dATP bound form) (**Figure 4A**) the inhibitory effect of RecU on RecA-mediated dATP hydrolysis was reduced, and that RecA dATPase activity is ∼eightfold less sensitive to RecU than its ATPase activity (**Figure 4A** and **Table 2** vs. Supplementary Table S3).

Preincubation of stoichiometric SsbA or SsbB concentrations (1 SSB/33-nt, 300 nM) with ssDNA extended the RecA lag phase (∼9 and ∼6 min, respectively) and affected the maximal dATP hydrolysis rates to different extents (*K*_cat_ ∼13.1 and ∼17.6 min^-1^, respectively) (**Figures 4B,C** and Supplementary Table S3; Manfredi et al., 2008; Yadav et al., 2012). We analyzed the non-catalytic inhibitory mechanism of RecU and SSB proteins (SsbA or SsbB) on RecA dATPase activity, to confirm whether SsbA or SsbB competes with RecU for ssDNA binding. Preincubation of ssDNA with stoichiometric SsbA or SsbB (1 SSB/33-nt) and saturating RecU (1 RecU/16-nt, 600 nM) concentrations reduced RecA-mediated dATP hydrolysis (*K*_cat_ ∼3.0 and ∼4.5 min^-1^, respectively). An excess of RecU (1 RecU/11-nt, 900 nM) and stoichiometric SSB concentrations (1 SSB/33-nt, 300 nM) were necessary to block RecA-catalyzed dATP hydrolysis (*K*_cat_ < 1 min^-1^) (**Figures 4B,C** and Supplementary Table S3).

When RecA was allowed to nucleate onto ssDNA for 5 min, followed by addition of increasing RecU concentrations (1 RecU/33- to 16-nt), dATP hydrolysis was unaffected for the first 8 min, after which it proceeded at a slower rate (**Figure 4D** and Supplementary Table S3). Addition of increasing RecU (1 RecU/33- to 16-nt, 300–600 nM) and fixed SsbA or SsbB concentrations to preformed RecA nucleoprotein filaments reduced RecA-mediated dATP hydrolysis (**Figure 4D** and Supplementary Table S3). These results confirmed that RecU and an SSB protein additively inhibit RecA nucleation and polymerization onto ssDNA.

### RecU Promotes Disassembly of Preformed RecA⋅ATP Nucleoprotein Filaments

**FIGURE 5:**
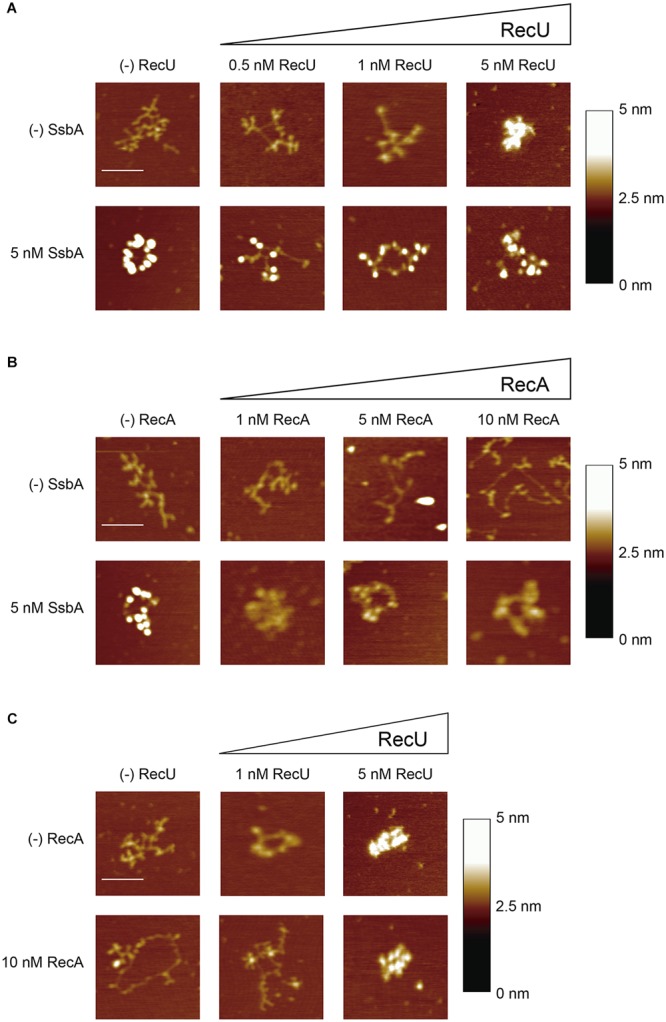
SsbA or RecU binding promotes disassembly of RecA-ssDNA complexes. **(A,B)** 3,199-nt ssDNA (0.1 nM in ssDNA molecules) was incubated with a fixed amount of SsbA and increasing RecU **(A)** or increasing RecA **(B)** concentrations in buffer C (10 min, 37°C). **(C)** ssDNA was preincubated with a fixed amount of RecA in buffer C (10 min, 37°C), followed by addition of increasing RecU concentrations, and the reaction was further incubated (10 min, 37°C). A fraction of the samples was deposited onto freshly cleaved mica and visualized by AFM. Each experiment was performed >3 times, with similar results. Bars, 100 nm.

To further define the mechanism by which RecU or SsbA inhibit RecA filament formation, we used AFM image analysis in dynamic RecA⋅ATP filament growth conditions. First, we analyzed the morphologies of the SsbA-ssDNA and RecU-ssDNA complexes in conditions for efficient RecA filament growth (10 mM Mg^2+^ and 5 mM ATP) (Cox, 2007; Cañas et al., 2008; Bell and Kowalczykowski, 2016). SsbA preferentially binds ssDNA with no secondary structures (Shereda et al., 2008). SsbA (1 SsbA/64-nt) extended the tangled or collapsed circular 3,199-nt ssDNA, resulting in a beads-on-a-string morphology (**Figure 5A**, *n* = ∼260). The beaded complexes were packed with an average of 19 ± 5 SsbA beads on the 3,199-nt ssDNA molecule (**Figure 5A**); this number did not increase significantly (22 ± 3) at higher SsbA:ssDNA ratios (data not shown). These data coincide with previous reports (Yadav et al., 2013) and suggest that SsbA does not disassemble stable secondary structures (Hamon et al., 2007).

RecU bound to ssDNA resulted in the formation of discrete globular structures (blobs) on DNA and led to compaction of the nucleoprotein complexes. At very low RecU concentrations (0.5 nM RecU, 1 RecU/640-nt), protein-ssDNA complexes were not observed. The collapsed ssDNA structure was partially disentangled by 1 RecU/320-nt (∼10 RecU/ssDNA molecule), and we observed protein-ssDNA complexes with an average of 6 ± 1 RecU blobs/ssDNA molecule (**Figure 5A**, *n* = ∼100). At a sub-saturating RecU concentration (5 nM, 1 RecU/64-nt), we found protein-ssDNA aggregates, which suggested that RecU–RecU interaction led to intramolecular DNA condensation (**Figure 5A**, *n* = ∼180).

When ssDNA was preincubated with stoichiometric SsbA concentrations (1 SsbA/64-nt, 5 nM) followed by increasing RecU concentrations (1 RecU/640- to 64-nt), we detected a smaller number of SsbA beaded structures and the protein-ssDNA complexes were diffuse. Compact RecU structures were not observed (**Figure 5A**, *n* = ∼100). These data are compatible with the presence of both proteins on the ssDNA. At the highest SsbA (5 nM) and RecU (5 nM) concentrations used, we did not detect the formation of RecU bridging structures (**Figure 5A**).

RecA is extremely slow to hydrolyze ATPγS, and the complete, contiguous RecA⋅ATPγS nucleoprotein filaments are poorly disassembled (reviewed in Cox, 2007; Bell and Kowalczykowski, 2016); in addition, ATP binding and its hydrolysis are crucial for dynamic formation and dissociation of RecA nucleoprotein filaments, with subsequent redistribution of bound RecA. To study the mechanism used by RecU to inhibit RecA nucleation and filament growth, experiments were performed in conditions of spontaneous RecA disassembly (RecA⋅ATP). The addition of RecA⋅ATP (1 RecA/320- to 32-nt, 1–10 nM) to ssDNA resulted in extended structures, which suggests that RecA bound to ssDNA might have melted most of the structure and then dissociated, leaving only some RecA-ssDNA (filament-like) structures (**Figure 5B**). In dynamic conditions, RecA (1 RecA/32-nt) facilitated formation of discrete short filament-like structures on extended circular complexes (**Figure 5B**, *n* = ∼150), which suggests that a fraction of RecA disassembled following interaction with the mica surface. Based on the size of the filament-like structures (∼12–29 nm) and at a pixel resolution of ∼2 nm (in a 512 pixel × 512 pixel/1 μm image), the filament might be composed of 20–50 RecA monomers bound to ssDNA and interrupted by long intervals of uncollapsed free ssDNA. This suggests that RecA assembles and then dissociates from the ssDNA, leaving it in an extended conformation.

When the ssDNA was preincubated with SsbA (1 SsbA/64-nt), followed by incubation with increasing RecA concentrations, the SsbA beaded structures did not undergo a noticeable change in morphology (1 RecA/64-nt, 5 nM) (**Figure 5B**), which suggests that RecA cannot displace SsbA from the ssDNA. This is consistent with an SsbA blockade of RecA ATPase activity (**Figure 2**). SSB*_Eco_* similarly outcompetes RecA*_Eco_* binding to ssDNA, as observed at single molecule resolution (Roy et al., 2009; Bell et al., 2012).

Similarly, RecA⋅ATP (1 RecA/32-nt) was preincubated with ssDNA, followed by incubation with increasing RecU concentrations (1 RecU/320- to 64-nt). In the presence of a limiting RecU concentration (1 RecU/320-nt, 1 nM), the ssDNA became tangled and RecA⋅ATP filament-like structures were not observed (**Figure 5C**, *n =* ∼150). At 1:2 RecU:RecA ratios, we detected compact RecU-DNA structures similar to those found when RecA⋅ATP was absent (**Figure 5C**, *n =* ∼150). These images are consistent with RecU binding to ssDNA once RecA has dissociated from the ssDNA after ATP hydrolysis. Alternatively, RecU inhibits RecA re-polymerization, resulting in its net depolymerization.

### DprA Protects RecA From the Inhibitory Effect of RecU

**FIGURE 6:**
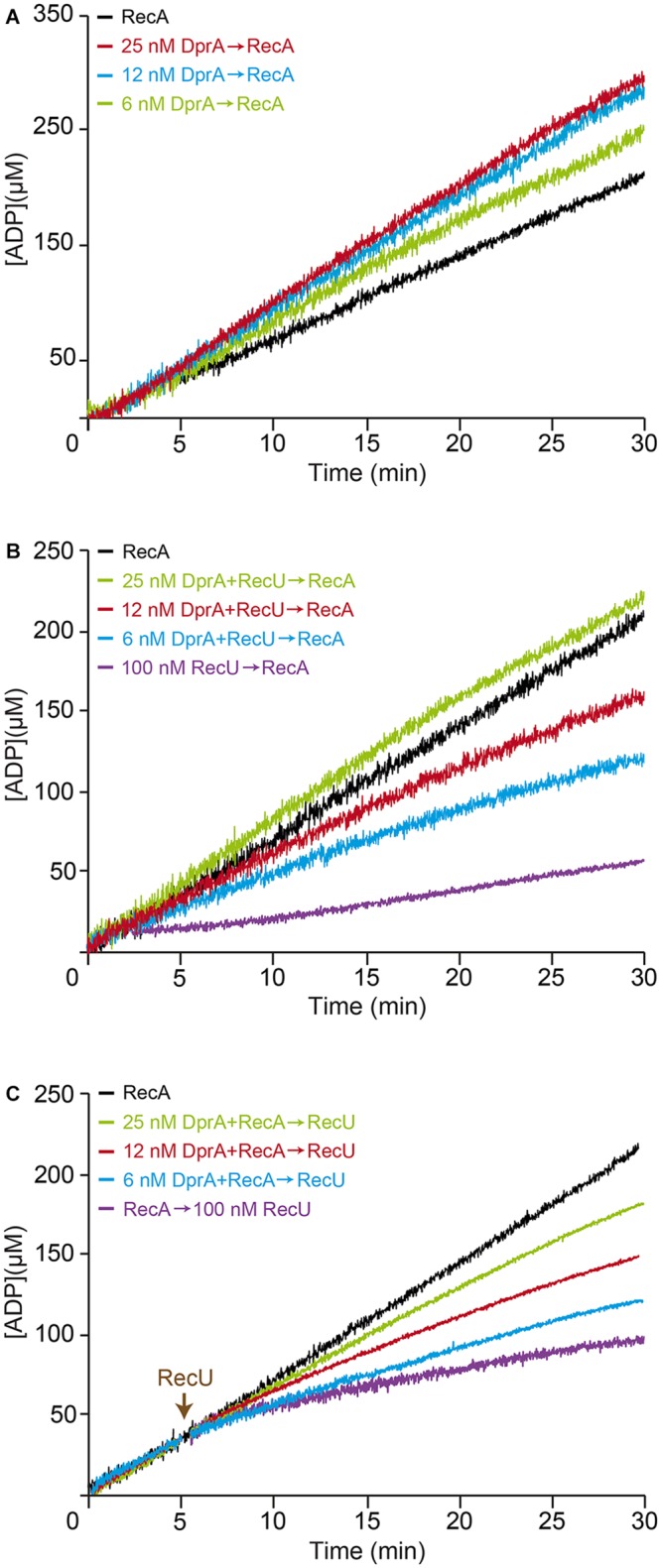
DprA antagonizes the inhibitory effect of RecU on RecA filament extension. **(A)** Circular 3,199-nt ssDNA (10 μM) was preincubated with increasing DprA concentrations (5 min, 37°C) in buffer B containing 5 mM ATP. RecA (0.8 μM) was added and ATPase activity was measured (30 min, 37°C). **(B)** ssDNA was preincubated with increasing DprA concentrations and a fixed RecU amount (100 nM) (5 min, 37°C), followed by the addition RecA. ATPase activity was then measured. **(C)** ssDNA was preincubated with a fixed amount of RecA and increasing DprA concentrations (5 min, 37°C), followed by a fixed amount of RecU (100 nM), and ATPase activity was measured. ATPase activity was measured from the time of RecA addition. Representative graphics are shown here and quantification of the results are expressed as the mean ± SEM of >3 independent experiments (see **Table 2**).

By interacting with RecA through the N-terminal α-helix and the DNA-binding domain, DprA binds and loads RecA⋅ATP onto ssDNA (Mortier-Barriere et al., 2007; Mirouze et al., 2013; Lisboa et al., 2014; Yadav et al., 2014). These RecA nucleoprotein filaments, however, cannot mediate DNA strand exchange, the two-component DprA-SsbA mediator is necessary to “activate” RecA⋅ATP to catalyze DNA strand exchange (Yadav et al., 2014; Carrasco et al., 2016). To test whether limiting DprA concentrations can reverse the negative effect of RecU on RecA assembly onto ssDNA, we studied the kinetics of RecA-mediated ATP hydrolysis in the presence of DprA and RecU. As few as 2–4 DprA dimers/ssDNA molecule (1 DprA/1,600- and 800-nt, 6 and 12 nM) stimulated RecA ATPase activity (*K*_cat_ ∼10.1 and ∼12.5 min^-1^) compared to conditions lacking DprA (*K*_cat_ ∼9.4 min^-1^) (**Figure 6A** and **Table 2**), confirming that the DprA recruits RecA onto ssDNA, and this effect increases the steady state of RecA bound to ssDNA. At 1 DprA/400-nt (24 nM), further RecA-mediated ATP hydrolysis was not stimulated (*K*_cat_ ∼12.5 min^-1^) (**Figure 6A** and **Table 2**), which implies that limiting amounts of DprA are necessary to recruit RecA onto ssDNA. This is consistent with the observation that sub-stoichiometric concentrations of *B. subtilis* DprA (1 DprA/66-nt, 150 nM) recruits RecA onto SsbA-coated ssDNA (Yadav et al., 2014), and that DprA*_Spn_* recruits even heterologous RecA*_Eco_* onto ssDNA (Mortier-Barriere et al., 2007).

A few DprA molecules (1 DprA/1,600- and 800-nt, 6–12 nM) partially reversed the negative effect of 100 nM RecU, on RecA-mediated ATP hydrolysis (*K*_cat_ ∼3.9 and ∼6.0 min^-1^, respectively) compared to controls with no DprA (*K*_cat_ ∼2.5 min^-1^) (**Figure 6B** and **Table 2**). When DprA and RecU were incubated together on the ssDNA at a ratio of 1:4 followed by the addition of RecA, DprA (1 DprA/400-nt) fully reversed the negative effect of RecU (1 RecU/100-nt) on RecA nucleation or filament extension onto ssDNA (*K*_cat_ ∼9.0 min^-1^) (**Figure 6B** and **Table 2**). Nucleation of RecA in the presence of increasing DprA concentrations (1 DprA/1,600-, 800-, and 400-nt) was also less sensitive to the inhibitory action of RecU (**Figure 6C** and **Table 2**). It is likely that limiting DprA reverses the action of RecU by increasing the steady state of RecA bound to the DNA.

### DprA Reverses the Negative Effect of RecU and SSB on the RecA ATPase

RecA efficiently nucleates on the DprA-ssDNA-SsbA or DprA-ssDNA-SsbB complexes, but only RecA nucleated on the DprA-ssDNA-SsbA complexes is active to catalyze DNA strand exchange (Yadav et al., 2014). To test whether DprA can reverse both RecU and SSB (SsbA or SsbB) inhibitors or if activated RecA can displace RecU from the ssDNA, we incubated RecU-ssDNA-SsbA or RecU-ssDNA-SsbB complexes with RecA⋅ATP and DprA.

**FIGURE 7:**
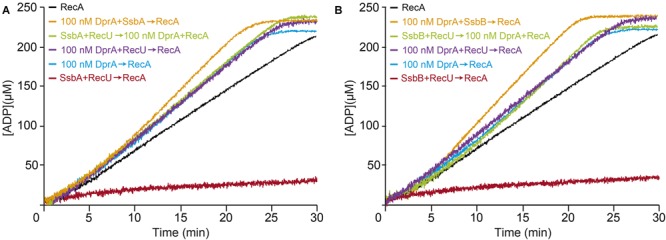
DprA facilitates RecA filament extension on the RecU-ssDNA-SsbA or RecU-ssDNA-SsbB complexes. **(A)** Circular 3,199-nt ssDNA (10 μM) was preincubated with SsbA (300 nM) and DprA or RecU (100 nM) (5 min, 37°C) in buffer B containing 5 mM ATP. RecA (0.8 μM) or RecA and DprA were added, and ATPase activity was measured (30 min, 37°C). Black line, control reaction corresponding to an ATPase assay in the absence of mediators (RecA and ssDNA only). **(B)** A similar reaction was performed adding SsbB (300 nM) was added instead of SsbA. The amount of ATP hydrolyzed was calculated. Representative graphics are shown here and quantification of the results are expressed as the mean ± SEM of >3 independent experiments (see **Table 2**).

The exact physiological concentrations of dimeric DprA in competent *B. subtilis* cells is unknown, but it should exceed the 1 μM concentration. DprA (100 nM) efficiently reversed the negative effect of RecU, SsbA (or SsbB) or both proteins on RecA nucleation and filament growth on the RecU-ssDNA-SsbA or RecU-ssDNA-SsbB complexes (**Figures 7A,B** and **Table 2**). It is likely that: (i) DprA alone is necessary and sufficient to stimulate RecA nucleation and filament growth on the RecU-ssDNA-SsbA or RecU-ssDNA-SsbB complexes; and (ii) activation of a RecA⋅ATP-mediated DNA strand exchange, via the concerted action of DprA and SsbA (Yadav et al., 2014), is dispensable to overcome the negative effect of RecU (**Figure 7**) and essential in the case of RecX (Le et al., 2017).

### DprA Modulates RecU Inhibition of RecA-Mediated Strand Exchange

**FIGURE 8:**
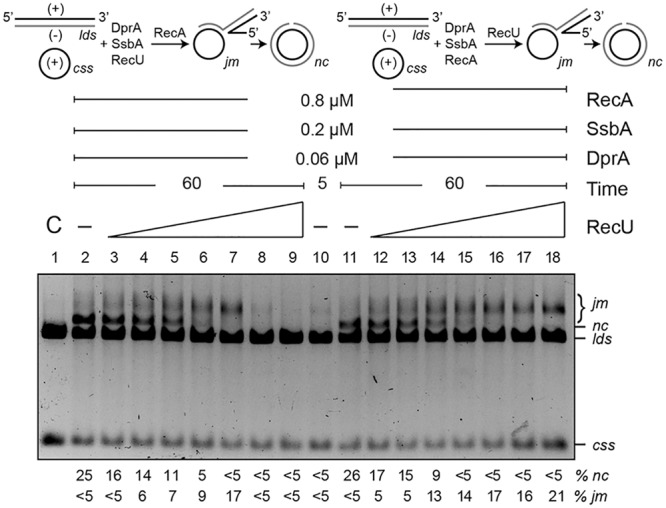
DprA antagonizes the inhibitory effect of RecU on DNA strand exchange. Top left, scheme of reactions performed on the left part of the gel (lanes 2–9). The *css* (+ strand in black) and the homologous *lds* (– in gray) substrates were preincubated with SsbA, DprA, RecU, and ATP (5 min, 37°C), after which RecA was added. Top right, scheme of the reactions performed on the right part of the gel (lanes 11–18). Here, DNA substrates were preincubated with SsbA, DprA, RecA, and ATP (5 min, 37°C), followed by RecU. The predicted intermediates (*jm*) and final products (*nc*) are illustrated. Homologous ssDNA (10 μM) and dsDNA (20 μM) were preincubated with SsbA, DprA and with variable concentrations of RecU (lanes 3–9; doubling from 6.2 to 400 nM) or a fixed RecA concentration (lanes 11–18) in buffer B containing 5 mM ATP (5 min, 37°C). A fixed RecA concentration (lanes 2–9) or variable amounts of RecU (lanes 12–18) were then added and the reaction was incubated (60 min, 37°C). Lane 1, DNA substrate controls (C); lanes 2 and 11, RecU was omitted, and in lane 10, the reaction was terminated after preincubation (5 min) without RecU. Reactions were resolved after deproteinization by 0.8% agarose gel electrophoresis. Band positions for *css, lds, cds, jm*, and *nc* are indicated. Bottom, recombination intermediates (*jm*) and products (*nc*) are expressed as the percentage in respect to the total substrate added. Results are shown as the mean ± 5% SEM of ≥3 independent experiments.

As revealed in the previous section, DprA is necessary to counteract the negative effect of RecU and SsbA on RecA⋅ATP nucleation and filament growth onto ssDNA. Previously, it has been shown that: (i) RecU (200 nM) inhibits RecA⋅dATP-mediated DNA strand exchange (Cañas et al., 2008); and (ii) the presence of DprA and SsbA are necessary and sufficient to activate RecA⋅ATP to catalyze DNA strand exchange (Yadav et al., 2014). To test whether DprA contributes to suppression of the negative effect of RecU, a three-strand exchange reaction in the presence of the two-component mediator DprA and SsbA and ATP was analyzed (**Figure 8**). In the presence of circular ssDNA (*css)*, homologous linear dsDNA (*lds)* and limiting concentrations of the two-component mediator (DprA-SsbA) RecA⋅ATP converted the substrate into intermediate (joint molecule, *jm*, <5%) and nicked circular (*nc*, ∼28%) products in a 60 min reaction (**Figure 8**, lane 2) as previously documented (Yadav et al., 2014; Carrasco et al., 2016). Limiting RecU concentrations (1:16 RecU:RecA molar ratio, 50 nM) added prior to RecA were sufficient to impair *nc* product accumulation in the presence of DprA and SsbA (**Figure 8**, lane 6). Addition of 100 nM RecU (or at a 1:8 RecU:RecA molar ratio) completely abolished RecA-mediated *nc* formation (**Figure 8**, lane 7). At higher RecU concentrations, RecA-mediated *jm* formation was abolished (**Figure 8**, lanes 8–9).

RecA⋅ATP nucleated and polymerized on the SsbA-ssDNA-DprA complexes was incubated with *lds* for 5 min, at which time traces of the substrate were converted to *jm* intermediates (**Figure 8**, lane 10). A variable amount of RecU was then added, and the reaction was incubated for 60 min (**Figure 8**, lanes 12–18). When RecU was omitted, RecA⋅ATP converted the substrate into *jm* intermediates (<5%) and *nc* products (∼28%) over a 60 min reaction period (**Figure 8**, lane 11). At RecU:RecA molar ratios of 1:16 (50 nM), *nc* products no longer accumulated. An excess of RecU (400 nM), which corresponds to a 1:2 RecU:RecA molar ratio, did not inhibit *jm* formation. It is likely that (i) RecA⋅ATP polymerized on the DprA-ssDNA-SsbA complexes and, after interaction with the homologous duplex, can promote accumulation of *jm* intermediates even at excess RecU concentrations (**Figure 8**, lanes 15–18), and (ii) RecU inhibits rather than reverses RecA-mediated DNA strand exchange.

## Discussion

Our genetic, biochemical, and biophysical results suggest that *B. subtilis* RecU positively contributes to plasmid transformation by inhibiting RecA activities. The central role of RecA in natural transformation is to assemble at the entry pole onto any incoming ssDNA to form a nucleoprotein filament, with the help of the DprA, SsbA (and SsbB) mediators. This filament has the unique capacity to search and find DNA sequences in the recipient dsDNA that are homologous to the ssDNA, resulting in homologous pairing and exchange of DNA strands (reviewed in Bell and Kowalczykowski, 2016), leading to chromosomal transformation (**Figure 1A**). If the incoming DNA is natural plasmid DNA, the RecA-mediated homology search is unproductive and the RecA nucleoprotein filaments are disassembled. RecU is crucial for plasmid transformation, but the absence of RecA partially supersedes the need for RecU (**Table 1**). Similarly, RecX positively contributes to plasmid transformation and negatively contributes to chromosomal transformation by regulating RecA activities (Le et al., 2017). We show that both negative RecA modulators are necessary for plasmid transformation. RecU and RecX blocked chromosomal and plasmid transformation, and that RecU modulates both chromosomal and plasmid transformation in the *recX* context (**Table 1**). The role of RecU and RecX during horizontal gene transfer via natural transformation is to modulate RecA activities, and to contribute to bacterial diversity. If the only role of the negative RecA modulators is to limit RecA filament growth, it could be predicted that competent *recX recU* cells should be at least partially proficient for plasmid transformation in the *recA* context. Unfortunately, this could not be tested because the introduction the Δ*recA* mutation into the chromosome by homologous recombination is prevented in a Δ*recX*Δ*recU* mutant strain.

The signal for RecA dissociation from heterologous ssDNA and its recycling is poorly understood. It is considered unlikely that a reduction in ATP levels (physiological concentration 10 mM, and ∼500-fold above RecA *K*_m_) would provide the signal for *in vivo* RecA filament disassembly from heterologous plasmid DNA. The results presented in this study and in a previous paper (Le et al., 2017) suggest that an unproductive homology search might be controlled by the negative RecA modulators, RecU and RecX (**Table 1**). RecU, which interacts with RecA (Carrasco et al., 2005; Cañas et al., 2008), blocked RecA-mediated ATP hydrolysis even at sub-stoichiometric concentrations (at a 1:8 RecU:RecA molar ratio) (**Figure 2**), suggesting that RecU does not have to interact with every RecA monomer on the filament to exert its inhibitory effect. SsbA and SsbB kinetically block RecA⋅ATP filament formation (Yadav et al., 2014). RecU and SsbA (or SsbB) additively inhibit RecA nucleation and filament growth onto ssDNA by a non-catalytic mechanism (**Figures 2C,D**, 4B,D). Our AFM data suggest that RecU-mediated RecA disassembly from preformed RecA filaments was faster than the normal filament disassembly.

At least four mechanisms for RecU inhibition of RecA nucleation and polymerization onto ssDNA can be envisioned: (a) RecU might block RecA filament extension by an active capping mechanism, as proposed earlier for RecX*_Eco_/*RecX (Ragone et al., 2008; Le et al., 2017), (b) RecU might actively dismantle the RecA filaments as shown by UvrD-like DNA translocases (as UvrD*/*PcrA, Srs2/PARI), (c) RecU might act as an antirecombinase by dissociating the RecA nucleoprotein filaments as proposed for the RuvAB*_Eco_* translocase (Adams et al., 1994; Iype et al., 1995), or (d) binding of RecU to ssDNA competes with RecA for ssDNA binding, and inhibits RecA nucleation and limits filament extension by directly interacting with RecA. We consider mechanism (a) to be unlikely, because nucleation of RecA⋅ATPγS onto the ssDNA can compete with the negative effect of RecU, but not of RecX (see **Figures 3C,D**). Several observations argue against mechanism (b): RecU neither hydrolyzes ATP nor translocates on ssDNA, and RecU inhibits RecA nucleation onto ssDNA, whereas PcrA or UvrD*_Eco_* actively disassemble RecA from ssDNA (Park et al., 2010; Fagerburg et al., 2012; Petrova et al., 2015). Furthermore, Srs2/PARI interacts with preformed Rad51 filaments and triggers ATP hydrolysis within the Rad51 filament, causing it to dissociate from ssDNA (Antony et al., 2009; Moldovan et al., 2012). The RecA–RecU interaction inhibits rather than stimulates the ATP hydrolysis rate. Mechanism (c) is unlikely, because the RuvAB*_Eco_* translocase leads to the rapid conversion of intermediates back to the original substrates (Adams et al., 1994; Iype et al., 1995), whereas RecU accumulates *jm* recombination intermediates (**Figure 8**), and the absence of RuvAB has a mild, if at all, effect on chromosomal and plasmid transformation (**Table 1**). Finally, mechanism (d) explains much of the data we obtained, which suggests that the ssDNA interaction with RecA⋅ATP and RecU or SsbA are mutually exclusive. Indeed, RecU formed blobs and SsbA formed beads on ssDNA (**Figure 5**).

**FIGURE 9:**
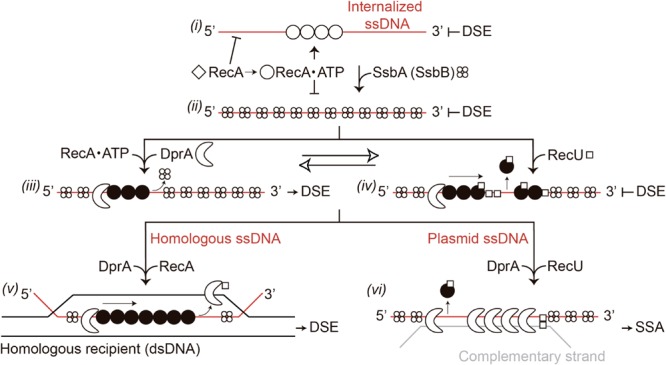
Model for RecA filament assembly on DprA-ssDNA-SsbA complexes in the presence of RecU. Apo RecA (empty diamonds) does not nucleate onto the internalized ssDNA. When ATP is present, RecA undergoes its first structural transition (empty circle). RecA-ATP can bind to incoming ssDNA and hydrolyze ATP, but cannot catalyze DNA strand exchange (DSE) (step *i*). When SsbA is present, RecA⋅ATP cannot nucleate on the SsbA-ssDNA complexes and cannot catalyze DNA strand exchange (step *ii*). Following interaction with DprA, SsbA recruits it onto the ssDNA. DprA in the DprA-ssDNA-SsbA complex interacts with and recruits RecA⋅ATP, to establish the 5′-end of the RecA filament. RecA undergoes its second structural transition (filled circle); and this active RecA assembles onto SsbA-coated ssDNA. Activated RecA remodels the SsbA-ssDNA complex to generate a DNA structure competent for SsbA displacement and RecA catalyzes DNA strand exchange in the presence of DprA-SsbA (step *iii*). RecU blocks RecA assembly on the ssDNA and promotes disassembly from the ssDNA (step *iv*). In the presence of homology with the recipient strand and a limiting concentration of DprA, it assists RecA⋅ATP assembly by displacing RecU to favor chromosomal transformation (step *v*). In the presence of plasmid ssDNA, DprA binds to plasmid ssDNA and, upon interaction with DprA-bound to a complementary incoming plasmid ssDNA (gray line), assists in the disassembly of RecA and RecU, and catalyzes single strand annealing (SSA) (step *vi*).

Based on previous reports (Yadav et al., 2014; Carrasco et al., 2015; Le et al., 2017) and the data shown here, we propose that RecA⋅ATP forms dynamic filaments on naked ssDNA, as visualized by AFM as an extension of the circular ssDNA relative to the compaction of the SsbA-coated ssDNA (**Figure 5B**), but cannot catalyze DNA strand exchange (**Figure 9**, step *i*) (Yadav et al., 2014). SsbA (or SsbA and SsbB) bound to ssDNA inhibits RecA⋅ATP nucleation and DNA strand exchange (**Figure 9**, step *ii*). SsbA (or SsbA and SsbB) bound to ssDNA interacts with and recruits DprA, which in turn interacts with and facilitates nucleation and subsequent RecA filament growth and RecA activation. Activated RecA⋅ATP can catalyze DNA strand exchange (**Figure 9**, step *iii*; Yadav et al., 2014; Carrasco et al., 2016). Once a region of homology is found, activated RecA⋅ATP forms a *jm* intermediate, followed by exchanges of the homologous strand on the supercoiled duplex chromosome by the incoming ssDNA, leading to chromosomal transformants (**Figures 1A, 9**, step *v*). We show that DprA and RecU contribute to plasmid transformation perhaps by regulating RecA activities, and this commitment step should be subjected to tight regulation. Addition of limiting DprA concentrations, at a 1:8 DprA:RecU molar ratio, reverses the negative effect of RecU (or SsbA and RecU) on RecA nucleation and filament growth on the SSB-coated ssDNA (**Figures 6, 7**). The interaction of the RecA nucleoprotein filament with the homologous duplex DNA protects it from RecU activity and favors chromosomal transformation (**Figure 9**, step *v*).

If activated RecA⋅ATP nucleates on a heterologous substrate (e.g., plasmid DNA), the search for homology between the internalized ssDNA and the host chromosome would be unproductive and energy-consuming. The anti-recombination effect of RecU favors the dismantling of the preformed RecA filaments and indirect elimination of the homology search between the internalized heterologous ssDNA and the supercoiled resident chromosome (**Figure 9**, step *iv*). This is consistent with the observation that the need for RecU (or RecX) for plasmid transformation is superseded in the Δ*recA* context (**Table 1**), and addition of RecU or SsbA to preformed RecA⋅ATP filaments enabled RecA disassembly by competing for binding to ssDNA, because the complexes observed by AFM are consistent with only RecU and SsbA binding (**Figures 5B,C**). The mechanism by which RecA assembly and disassembly are regulated by the DprA and RecU proteins during natural transformation is poorly understood. We suggest that RecA depolymerization by RecU, and a DprA–DprA interaction reverses the role of DprA as a mediator and favors its single-strand annealing (SSA) activity for plasmid transformation.

We propose that DprA has four activities during natural transformation: (i) to facilitate RecA nucleation and filament growth on SsbA-coated ssDNA and to help RecA to mediate DNA strand exchange (crucial for chromosomal transformation) (**Figures 6, 7**); (ii) to inhibit RecA nucleation on the ssDNA when present in an excess with respect to RecA (e.g., at 1.1:1–1.5:1 DprA:RecA molar ratio) (Yadav et al., 2014); (iii) to mediate strand annealing of complementary strands coated by SsbA or SsbB during plasmid transformation (Yadav et al., 2013, 2014); and (iv) to counteract RecU-mediated inhibition of RecA nucleation and filament growth (chromosomal transformation), but in concert with RecU to promote plasmid transformation. We hypothesize that during plasmid transformation, RecU bound to ssDNA might facilitate DprA loading (**Figure 9**, steps *iv, vi*), perhaps by a direct protein-protein interaction (see Annex 1 in Supplementary Materials and Supplementary Figure S1). The balance of the RecA filament stabilizing activity of DprA and destabilizing activity of RecU would be established before DprA switches from its RecA mediator to its strands annealing activity (**Figure 9**, step *iii* vs. **Figure 9**, step *iv*). DprA, at low DprA:RecU:RecA molar ratios (1:16:64–1:8:32), works as a mediator to antagonize the negative effect of RecU, facilitating RecA-mediated nucleation and filament growth onto ssDNA, inhibiting plasmid transformation (**Figures 6B,C**, 9, step *v*). At least two mechanisms can be conceived for the antagonistic effects of DprA on RecU-mediated inhibition of RecA filament formation. First, DprA bound to the SsbA- (or SsbA and SsbB)-coated ssDNA region enables RecA loading onto ssDNA and antagonizes the RecU interaction with RecA. Second, the two-component mediator (DprA-SsbA) activates RecA to directly antagonize RecU. The first mechanism assigns more importance to DprA interaction with RecA and RecU, whereas the latter gives greater weight to RecA activation via DprA and SsbA. We ruled out the second mechanism, because DprA (at a 1:8:32 DprA:RecU:RecA molar ratio) is sufficient to reverse RecU inhibition of the RecA ATPase (**Figure 6**).

DprA bound to the RecU-generated discontinuities in the pre-assembled RecA filament and to another DprA in the complementary plasmid strand might undergo a transition, leading to DprA assembly as a nucleoprotein complex (Mortier-Barriere et al., 2007; Yadav et al., 2013; Dwivedi et al., 2015). This nucleoprotein complex, in concert with RecU, promotes RecA depolymerization from the heterologous plasmid DNA and favors plasmid transformation (**Figure 9**, step *vi*). This is consistent with the observation that DprA at a 1:1.5 DprA:RecA molar ratio inhibits rather than stimulates RecA nucleation and filament growth on the ssDNA (Yadav et al., 2013, 2014). DprA bound to both SsbA- (or SsbB)-coated complementary strands anneals them to reconstitute a circular plasmid replicon (**Figure 9**, step *vi*; Yadav et al., 2013). RecU, which also mediates DNA pairing (Ayora et al., 2004; Carrasco et al., 2005), might help DprA to catalyze single-strand annealing (**Figure 9**, step *vi*). DprA engaged in single-strand annealing might in turn allow RecU to further inhibit RecA filament formation on the heterologous plasmid ssDNA. This is consistent with the observation that plasmid transformation is reduced to a similar extent in competent Δ*recU* Δ*dprA* and Δ*dprA* cells (**Table 1**). Many details of the impact of DprA and RecU proteins in genetic recombination may not become apparent until their genes are studied in the absence of redundant and/or complementary protein functions.

## Materials and Methods

### Strains and Plasmids

*Bacillus subtilis* BG214 cells (*rec****^+^***) and its isogenic marker-free derivative lacking Δ*recA*,Δ*recU*, Δ*ruvAB*,Δ*dprA*, Δ*recU* Δ*recA*, or Δ*dprA* Δ*recA* have been reported (Kidane et al., 2012) and are listed in **Table 1**. The gene to be characterized (Δ*dprA* or Δ*recX*) was deleted by gene replacement with the *six*-*cat*-*six* (SCS) cassette flanked by homology up- and downstream in the Δ*recU* context, as described (Torres et al., 2017). The SCS cassette is composed of two directly oriented β-recombinase cognate sites (*six* sites) and the *cat* gene, which confers chloramphenicol resistance (Cm^R^). Natural competent cells were transformed with the SCS cassette flanked by homologous regions to the gene to be deleted with selection for Cm^R^. Integration of the SCS cassette, through double crossover recombination, replaced the *dprA* or *recX* gene. This was followed by β site-specific recombinase-mediated excision between the two directly oriented *six* sites, leading to deletion of the *cat* gene and one *six* site (marker-free). The outcome of this strategy is that the gene to be characterized is deleted and is replaced by a single *six* site (Torres et al., 2017).

*Escherichia coli* BL21(DE3) [pLysS] cells bearing the pCB568 (*recU*), pCB722 (*ssbA*), pCB777 (*ssbB*), pCB888 (*dprA*), or pCB936 (*recX* gene) plasmids were used to overproduce their respective proteins (Carrasco et al., 2008; Yadav et al., 2012, 2013; Le et al., 2017). *Bacillus subtilis* BG214 cells bearing pBT61 (*recA* gene) was used to overproduce RecA (Gassel and Alonso, 1989). The 3,199-base pair (bp) pGEM3 Zf(+) was used as a source of dsDNA and ssDNA (Promega Biotech).

Unless stated otherwise, the indicated genes and products are from *B. subtilis* origin. The nomenclature used to denote the origin of proteins from other bacteria is based on the bacterial genus and species (e.g., *E. coli* RecA is referred to as RecA*_Eco_*).

### Natural Transformation

For DNA transformation experiments, competent *B. subtilis* cells were transformed with 100 ng of SB19 chromosomal DNA to *met****^+^*** (chromosomal transformation) or of pUB110 plasmid DNA to neomycin resistance (Nm^R^) (plasmid transformation). Chromosomal transformants were plated on minimal medium lacking methionine, and plasmid transformants on LB agar plates containing Nm (5 μg⋅ml^-1^) (Alonso et al., 1988).

### Enzymes, Reagents, Protein, and DNA Purification

All chemicals used were analytical grade. Isopropyl β-D-1-thiogalactopyranoside (IPTG) was from Calbiochem; DNA restriction enzymes and DNA ligase from Biolabs, and polyethyleneimine, dithiothreitol (DTT), ATP, dATP, and the poorly hydrolyzable ATP analog ATPγS from Sigma. DEAE-, Q-, and SP-Sepharose were from GE Healthcare, hydroxyapatite from Bio-Rad, and phosphocellulose from Whatman.

*Bacillus subtilis* SsbA, SsbB, RecU, DprA, RecX, and RecA proteins were overexpressed and purified (Carrasco et al., 2005; Manfredi et al., 2008; Yadav et al., 2012, 2013; Le et al., 2017). All proteins were purified to >98% homogeneity, and their molar extinction coefficients at 280 nm were calculated as 11,400, 13,000, 23,900, 45,500, 16,400, and 15,200 M^-1^ cm^-1^, respectively (Carrasco et al., 2005). Protein concentrations were determined using these extinction coefficients. RecA and RecX are expressed as moles of monomeric, RecU and DprA as dimeric, and SsbA and SsbB as tetrameric proteins.

Duplex and ssDNA from pGEM3 Zf(+) were purified (Carrasco et al., 2005). DNA concentrations were established using the molar extinction coefficients at 260 nm of 8,780 and 6,500 M^-1^ cm^-1^ for ssDNA and dsDNA, respectively. Protein concentrations are expressed in the text as stoichiometric ratios relative to ssDNA, which is expressed as moles of nt, whereas figure legends give the molar concentrations of proteins and ssDNA/dsDNA.

Protein crosslinking by *bis*-disuccinimidyl suberate (DSS) was used to study protein–protein interactions (Carrasco et al., 2005). A constant amount of RecU or a variant lacking the first 32 RecU residues (termed RecUΔ32) and DprA were mixed in buffer A [50 mM Tris–HCl (pH 7.5), 50 mM NaCl, 10 mM MgOAc, 1 mM DTT, 5% glycerol]. DSS was added to a final concentration of 5 μM; after incubation (15 min, 37°C), reactions were stopped according to manufacturer’s instructions (Thermo Fisher Scientific). Rabbit polyclonal anti-RecU or -DprA antibodies were obtained as described (Cañas et al., 2008). For Western blotting, proteins were separated by 10% SDS-PAGE and blots were probed with anti-RecU or -DprA antibodies.

### RecA (d)ATP Hydrolysis Assays

The ssDNA-dependent ATP or dATP [(d)ATP] hydrolysis activity of the RecA protein was assayed via a NAD/NADH coupled spectrophotometric enzyme assay (Hobbs et al., 2007). In optimal conditions for the RecA ATPase, excess RecU (1 RecU dimer/11-nt), SsbA, or SsbB (1 SSB tetramer/15-nt) concentrations did not hydrolyze ATP (Carrasco et al., 2005; Yadav et al., 2012).

The rate of ssDNA-dependent RecA-mediated (d)ATP hydrolysis (catalytic constant, *K*_cat_) and the time needed to reach a steady-state (d)ATP hydrolysis rate (lag time) were measured in buffer B (50 mM Tris–HCl [pH 7.5], 80 mM NaCl, 10 mM MgOAc, 50 μg⋅ml^-1^ BSA, 1 mM DTT, 5% glycerol) containing 5 mM (d)ATP (30 min, 37°C) (Yadav et al., 2012). In our experimental conditions, the Mg^2+^ ion is in excess of that needed to chelate available ATP (5 mM), to maintain RecA in its active state (Carrasco et al., 2008). The order of addition of 3,199-nt pGEM3 Zf(+) ssDNA (10 μM in nt) and purified proteins are indicated in the text. When indicated, 3-WJ DNA was also added. The 16-M (5′-GACGCTGCCGAATTCTACCAGTGCCTTGCT AGGACATCAGTCCTTACCTGCAG GTTCAC-3′), 17-M (5′-G GGTGAACCTGCGGTAAGGGGCTGCTCATCGTAGGTTAGT TGGTAGAATTCGGCAGC-3′), and 19-M (5′-TAAGAGCAAG ATGTTCCTCAACTGATGTCCTAGCAAGGCAC-3′) oligonu-cleotides were hybridized to form the 3-WJ DNA, as described (Zecchi et al., 2012).

Data obtained from ATP/dATP hydrolysis were converted to (ADP/dATP) and plotted as a function of time (Yadav et al., 2012). The lag time, which represents the delay in reaction progress relative to a theoretical reaction curve that lacks a lag time, was derived from the time intercept of a linear regression line fit to the steady state portion of data in (d)ATP hydrolysis assays (Hobbs et al., 2007; Yadav et al., 2012).

### RecA-Mediated DNA Strand Exchange

The KpnI-cleaved 3,199-bp pGEM3 Zf(+) dsDNA (20 μM in nt) and the homologous circular 3,199-nt ssDNA (10 μM in nt) were preincubated with indicated concentrations of SsbA, DprA and increasing concentrations of RecU (6.2–400 nM by doubling the protein concentration) or fixed RecA in buffer B containing 5 mM ATP (5 min, 37°C). A fixed RecA or variable RecU concentration was then added, and the reaction incubated (60 min, 37°C). An ATP regeneration system (8 U ml^-1^ creatine phosphokinase and 8 mM phosphocreatine) was included in the recombination reaction. After reaction, samples were deproteinized and fractionated by 0.8% agarose gel electrophoresis with ethidium bromide (Ayora et al., 2002a,b). Signals of DNA substrates, intermediates and products were quantified from gels using a Gel Doc system (Bio-Rad) (Carrasco et al., 2008).

### Single Molecule Analyzes

Formation of SsbA-, RecU-, RecA-ssDNA complexes, alone, or in combination, was measured by atomic force microscopy (AFM) in buffer C [5 mM HEPES (pH 7.5), 65 mM NaCl, 5 mM MgCl_2_, 1 mM DTT, 5% glycerol] containing 5 mM ATP. The solution was then diluted in buffer C and spotted onto freshly cleaved mica. The mica was pretreated with 5 mM spermidine (10 min, room temperature), washed several times with MilliQ water, and dried under a nitrogen stream. The circular 3,199-nt pGEM3 Zf(+) ssDNA (0.1 nM in ssDNA molecules) was incubated with the indicated concentration of proteins (10 min, 37°C). A fraction of the sample was deposited on a mica surface, and processed as described (Sanchez et al., 2008). AFM observations were performed with a Nanoscope IIIa (Digital Instruments) in air using the tapping mode. The cantilever (OMCL-AC160TS-W2, Olympus) was 160 μm long with a 33–62 N/m spring constant.

The scanning frequency was 2–3 Hz, and images were captured with the height mode in a 512 pixels × 512 pixels format. Images were plane-fitted and flattened by the computer program accompanying the imaging module. The “tip effect” was compensated for using the apparent size of DNA as a reference. Volume was analyzed using Image SXM 2.01 software^1^ (Barrett, 2008), and histograms and Gaussian curves were drawn using Origin 6 software (Deschenes and Vanden Bout, 2000). Image processing of the topographs and height measurements was performed as described (Pratto et al., 2009).

## Author Contributions

JA conceived and supervised the study and wrote the manuscript. ES, BC, and JA designed the experiments and analyzed the data. ES, BC, JG, and KT performed the experiments and contributed to writing the paper.

## Conflict of Interest Statement

The authors declare that the research was conducted in the absence of any commercial or financial relationships that could be construed as a potential conflict of interest.

## Footnotes

1 https://www.liverpool.ac.uk/~sdb/ImageSXM
