# Supplementary material for: RecA Regulation by RecU and DprA During Bacillus subtilis Natural Plasmid Transformation
Source: Front Microbiol. 2018 Jul 11;9:1514. doi: 10.3389/fmicb.2018.01514 (PMC6050356; doi:10.3389/fmicb.2018.01514)
Supplement: Supplementary file 1 [file Presentation_1.pdf]

Supplementary Material

RecA Regulation by RecU and DprA During *Bacillus subtilis* Natural Plasmid Transformation

Ester Serrano[1], Begoña Carrasco[1], Jamie L. Gilmore[2], Kunio Takeyasu[2], and Juan C. Alonso[1,*]

[1]Laboratory 214, Department of Microbial Biotechnology, Centro Nacional de Biotecnología, CNB-CSIC, 28049 Madrid, Spain.

[2] *Graduate School of Biostudies, Yoshida-Konoe-cho, Sakyo-ku, Kyoto 606-8501, Japan*

***Correspondence:**
Juan C. Alonso
jcalonso@cnb.csic.es

1 Supplementary Data

Annex 1. DprA interacts with RecU

RecU is epistatic to DprA during plasmid transformation (Table 1). *In vivo* co-localization studies suggested that RecU and DprA interact (Kidane et al., 2009), but this interaction has not been shown *in vitro*. We used limiting concentrations of the crosslinking agent *bis*disuccinimidyl suberate (DSS), which has a spacer arm length of 11.4 Å, to test whether DprA physically interacts with RecU. In the absence of crosslinker, DprA (predicted molecular mass of 32.7 kDa) was resolved as a major band of ~33 kDa (D) and a very minor ~66 kDa band (D_2) (Supplemental Figure S1a-b, lane 1), and RecU (23.9 kDa predicted) was resolved as a major ~24 kDa (U) and a minor ~48 kDa (U_2) band (in SDS-PAGE with limiting DTT concentrations to avoid disulphide bond breakage), as highlighted with polyclonal anti-DprA and -RecU antibodies, respectively (Figure S1a, lane 4). DprA and RecU crystallize as dimers, but tetramers are also found (McGregor et al., 2005; Mortier-Barriere et al., 2007). After limiting DSS crosslinking, we detected DprA bands of ~33 and ~66 kDa in agreement with a dimer, and diffuse bands of larger molecular mass (Supplemental Figure S1a-b, lane 2); whereas RecU showed bands of ~24, ~48 (U_2, dimer), and in a very minor proportion, ~72 (U_3) and even ~96 kDa (U_4) (Figure S1a, lane 5). When DprA was incubated with RecU and DSS, anti-DprA antibodies showed an additional minor band of ~57 kDa (DU), with a molecular mass equivalent to one DprA monomer crosslinked to one RecU (Figure S1a, lane 3). This band was also seen when the membrane was developed with anti-RecU antibodies (Figure S1a, lane 6; arrow). Such crosslinked products were not observed when DprA or RecU was replaced by a 20-fold excess of bovine serum albumin (BSA) (not shown).

A RecU variant that lacks the flexible N-terminal 32 residues (RecUΔ1–32) does not interact with the RuvB translocase, although it modulates RecA activities (Carrasco et al., 2009). When DprA was incubated with th2 RecUΔ1-32 variant and DSS, the Western blot using anti-DprA or -RecU antibodies showed a minor band of ~54 kDa (Figure S1b, lanes 3 and 6; DΔ32, arrow), which suggests that this

band is composed of one RecUΔ1-32 and one DprA monomer, and that RecUΔ1-32 is still able to interact with DprA. The addition of ssDNA yielded similar results (not shown). It is likely that RecU and DprA interact transiently, even in the absence of ssDNA, and that different RecU domains interact with DprA and with RuvB.

2 Supplementary Figures and Tables

Table S1

RecA-mediated ATP hydrolysis in the presence of RecU and 3-WJ DNA

Proteins[a]	Lag time[a] (min)	k_{cat} min^{-1}[a]
RecA (1 RecA/12-nt)[b]	<1	9.4 ± 0.2
100 nM 3-WJ DNA → RecA	<1	9.7 ± 0.4
400 nM 3-WJ DNA → RecA	<1	10.1 ± 0.6
RecU/100-nt → RecA	~1	2.4 ± 0.3
RecU/100-nt + RecA → 100 nM 3-WJ DNA	~1	2.9 ± 0.4
RecU/100-nt + RecA → 200 nM 3-WJ DNA	<1	3.5 ± 0.3
RecU/100-nt + RecA → 400 nM 3-WJ DNA	<1	5.4 ± 0.3
RecA → RecU/100-nt	-	2.5 ± 0.4
100 nM 3-WJ DNA + RecA → RecU/100-nt	-	2.7 ± 0.4
200 nM 3-WJ DNA + RecA → RecU/100-nt	-	4.0 ± 0.3
400 nM 3-WJ DNA + RecA → RecU/100-nt	-	6.3 ± 0.2

[a]Rates of RecA-mediated ATP hydrolysis and nucleation lag times with ssDNA as effector were measured (see Experimental procedures). [b]RecA-mediated ATP hydrolysis rates and lag times were reported elsewhere (Carrasco et al., 2008; Manfredi et al., 2008; Yadav et al., 2012), and determined here for direct comparison. The steady state kinetic parameters for RecA (1 RecA/12-nt) in the presence

of RecU (1 RecU/100-nt) or increasing concentrations of 3-WJ DNA (100 to 400 nM) were derived from more than three independent experiments like those in Fig. 2A-B; results are shown as mean ± SEM. The + symbol indicates that proteins were preincubated, and arrows indicate the order of protein or 3-WJ DNA addition. -, not applicable.

Table S2

Rates of RecA-mediated ATP hydrolysis in the presence of RecU or RecX

Proteins[a]	ATPγS (in μM)	Lag time[a] (min)	k_{cat} min^{-1a}
RecA (1 RecA/12-nt)[b]	-	<1	9.4 ± 0.2
RecA	3	<1	8.9 ± 0.4
RecA	24	<1	4.8 ± 0.3
RecA → RecU/100-nt	-	<1	2.5 ± 0.3
RecA → RecU/100-nt	3	<1	2.7 ± 0.2
RecA → RecU/100-nt	24	<1	5.2 ± 0.4
RecA → RecX/100-nt	-	-	2.1 ± 0.2
RecA → RecX/100-nt	3	-	2.1 ± 0.1
RecA → RecX/100-nt	24	-	2.0 ± 0.2

[a]Rates of RecA-mediated ATP hydrolysis and nucleation lag times were measured (see Experimental procedures). [b]RecA-mediated ATP hydrolysis rates were reported elsewhere (Carrasco et al., 2008; Manfredi et al., 2008; Yadav et al., 2012), and determined here for direct comparison. The steady state kinetic parameters for RecA (1 RecA/12-nt) in the presence of RecU (1 RecU/100-nt) or RecX (1 RecX/100 nt) and different ATPγS concentrations (3 and 24 μM) were derived from more than three independent experiments like those in Fig. 2C-D; results are shown as mean ± SEM. Arrows denote the order of protein addition to reactions. -, not applicable.

Table S3

Rates of ssDNA-dependent dATP hydrolysis and lag time measurements in the presence of RecU and SSB proteins

Proteins[a]	Lag time[a] (min)	k_{cat} min^{-1}[a]
RecA (1 RecA/12-nt)[b]	4 ± 0.5	17.8 ± 0.4
SsbA/33-nt → RecA[b]	~9	13.1 ± 0.3
SsbB/33-nt → RecA[b]	~6	17.6 ± 0.3
RecU/33-nt → RecA	~9	12.5 ± 0.2
RecU/16-nt → RecA	~13	10.2 ± 0.2
RecU/11-nt → RecA	-	<1
RecA → RecU/33-nt	-	10.5 ± 0.3
RecA → RecU/16-nt	-	8.9 ± 0.3
RecA → RecU/11-nt	-	<1
RecU/33-nt + SsbA/33-nt → RecA	~9	8.1 ± 0.3
RecU/33-nt + SsbB/33-nt → RecA	~9	11.3 ± 0.2
SsbA/33-nt + RecA → RecU/33-nt	~4	8.3 ± 0.3
SsbB/33-nt + RecA → RecU/33-nt	~2	12.5 ± 0.3
RecU/16-nt + SsbA/33-nt → RecA	~9	3.0 ± 0.2
RecU/16-nt + SsbB/33-nt → RecA	~5	4.5 ± 0.2
SsbA/33-nt + RecA → RecU/16-nt	<1	4.1 ± 0.3

SsbB/33-nt + RecA → RecU/16-nt	<1	4.2 ± 0.3
RecU/11-nt + SsbA/33-nt → RecA	-	<1
RecU/11-nt + SsbB/33-nt → RecA	-	<1

[a]Rates of RecA-mediated dATP hydrolysis and nucleation lag times were measured (see Experimental procedures). [b]RecA-mediated dATP hydrolysis and lag times were reported elsewhere (Carrasco et al., 2008; Manfredi et al., 2008; Yadav et al., 2012), and determined here for direct comparison. The steady state kinetic parameters for RecA (1 RecA/12-nt) were derived from more than three independent experiments like those in Fig. 3*;* results are shown as mean ± SEM. The + symbol indicates that proteins were preincubated, and arrows indicate the order of protein addition. Concentrations of all other components are listed individually. -, not applicable.

2.1 Supplementary Figures

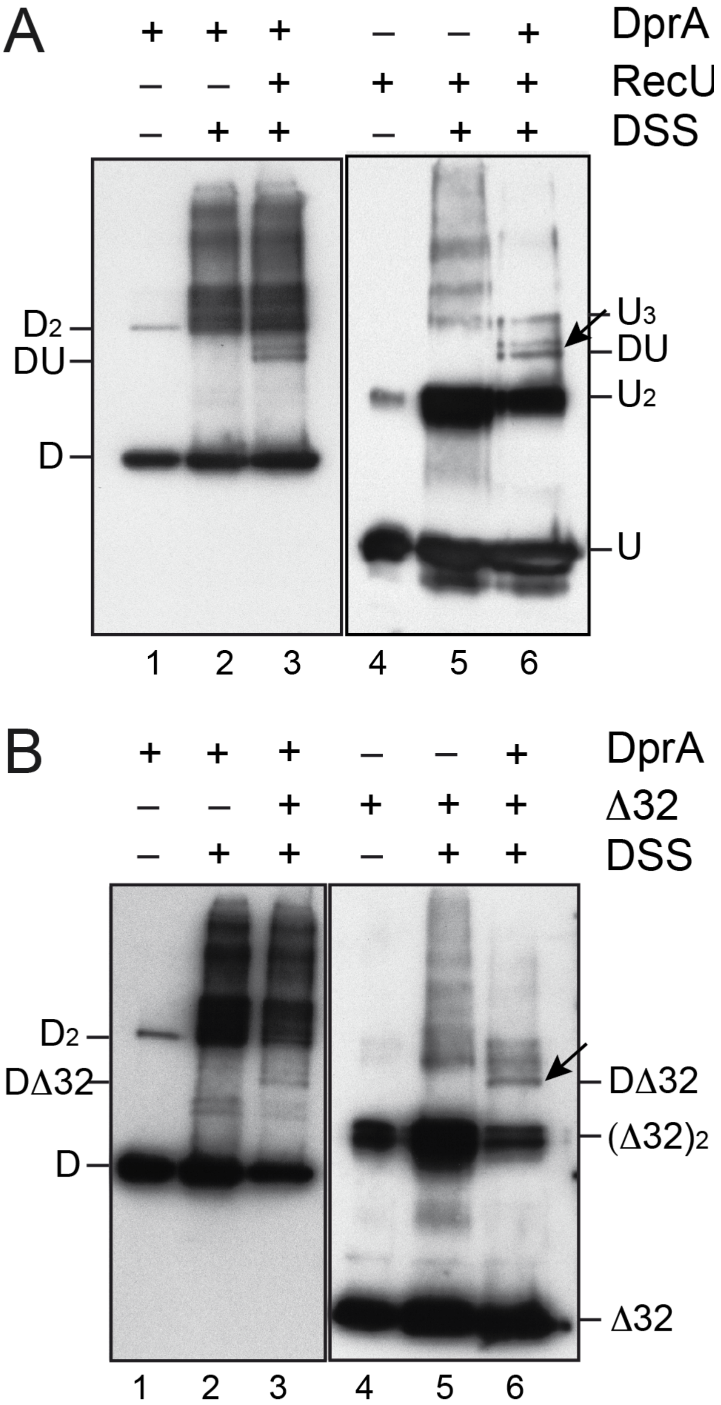

Figure S1. RecU interacts with DprA. RecU (*A*) or RecUΔ1-32 (*B*) (3 μM) was incubated with DprA (*A* and *B*) (4.5 μM each) in the presence of 50 nM DSS (15 min, 37ºC) in buffer A, followed by immunoblotting. The crosslinked proteins were detected using anti-DprA (lanes 1-3) or anti-RecU antibodies (lanes 4-6). The 57 kDa (DprA-RecU, DU) (*A*) or 54 kDa (DprA-RecUΔ1-32, DΔ32) (*B*) bands are indicated by arrows. The + and – symbols indicate the presence or absence of the indicated protein and/or crosslinking agent.
